# Revealing the Intricate Structure of Surface Phases of Methanol on In_2_O_3_(111)

**DOI:** 10.1021/acs.jpcc.5c07043

**Published:** 2026-01-28

**Authors:** Andreas Ziegler, Chiara I. Wagner, Hao Chen, Matthias A. Blatnik, Alexander Wolfram, Anne Brandmeier, Zdeněk Jakub, Michele Riva, Jiri Pavelec, Michael Schmid, Ulrike Diebold, Bernd Meyer, Margareta Wagner

**Affiliations:** † Interdisciplinary Center for Molecular Materials (ICMM) and Computer Chemistry Center (CCC), Friedrich-Alexander-Universität Erlangen-Nürnberg (FAU), 91052 Erlangen, Germany; ‡ Institute of Applied Physics, TU Wien, 1040 Vienna, Austria; § Central European Institute of Technology (CEITEC), Brno University of Technology, 61200 Brno, Czech Republic; ∥ Lehrstuhl für Physikalische Chemie II, Friedrich-Alexander-Universität Erlangen-Nürnberg (FAU), 91058 Erlangen, Germany

## Abstract

Research on sustainable energy has intensified to reduce greenhouse gas emissions, especially CO_2_. One promising strategy is the catalytic reduction of CO_2_ to methanol, and indium oxide (In_2_O_3_) has emerged as a highly efficient catalyst, with high turnover rates and selectivity. This work investigates methanol, the end product of CO_2_ reduction, and its interaction with the In_2_O_3_(111) surface. Utilizing an ultrahigh vacuum (UHV) environment, this study combines temperature-programmed desorption (TPD), X-ray photoelectron spectroscopy (XPS), noncontact atomic force microscopy (nc-AFM), scanning tunneling microscopy (STM), and density functional theory (DFT) calculations. The coverages investigated range from 1 to 12 methanol molecules per unit cell. The results are compared to water adsorption on In_2_O_3_(111), as the chemical behavior of both molecules is similar in many respects. At low coverage, the adsorption patterns and interactions with the In_2_O_3_(111) surface mirror those seen with water, including dissociative and molecular adsorption. The first three methanol molecules dissociate at specific sites within the surface unit cell, while molecular adsorption becomes favored for subsequent molecules at temperatures below 300 K. At the highest coverage (before multilayer adsorption) methanol and water exhibit distinct structures due to their differing hydrogen bonding capabilities.

## Graphical abstract

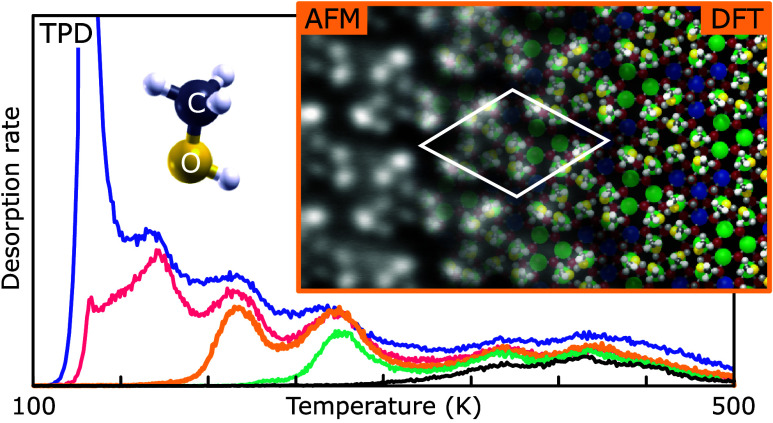

## Introduction

The need to mitigate greenhouse gas emissions, particularly carbon dioxide (CO_2_), has intensified research into sustainable energy solutions. Among the various strategies being explored, the catalytic reduction of CO_2_ to valuable chemicals, such as methanol, has gained considerable attention.^1–3^ Methanol is a versatile molecule that can serve as a renewable fuel, energy carrier, and precursor for the synthesis of numerous chemical products. While the focus of fundamental investigations is usually on the interaction of CO_2_ with potential catalyst materials and the charge transfer into the molecule at the active site, elucidating how reaction intermediates or the desired end product interact with the catalyst and its support is also relevant.

Indium oxide has emerged as a promising catalyst for CO_2_ reduction due to its high selectivity, thus enhancing the overall efficiency of methanol production.^4–7^ To utilize the full potential of In_2_O_3_ and optimize its catalytic activity, it is essential to understand the processes occurring at the catalyst surface at the atomic level. The (111) surface is the most stable facet of In_2_O_3_ and due to its prevalence in powder samples it is often associated with the highly selective catalytic activity of this material.^8–13^ Due to its large surface unit cell with 3-fold symmetry, In_2_O_3_(111) offers a variety of inequivalent undercoordinated indium and oxygen atoms with different properties such as different proton affinity.^14^ Figure 1a presents the atomic structure of the relaxed bulk-terminated surface. The symmetry-equivalent 5- and 6-fold coordinated In atoms and 3-fold coordinated O atoms are labeled as In­(a)–In­(f) and O­(α)–O­(δ), respectively; the three 3-fold symmetry axes are indicated as A, B, and C. Note that we place the unit cell such that its corners are in the high-symmetry point B, the center of the 3-fold star of 6-fold In atoms. In constant-height nc-AFM images of the stoichiometric surface, as provided in Figure 1b, the presence of all surface oxygen atoms O­(α)–O­(δ) is evident. Surface oxygen vacancies are not expected on this surface.^15^

**Figure 1:**
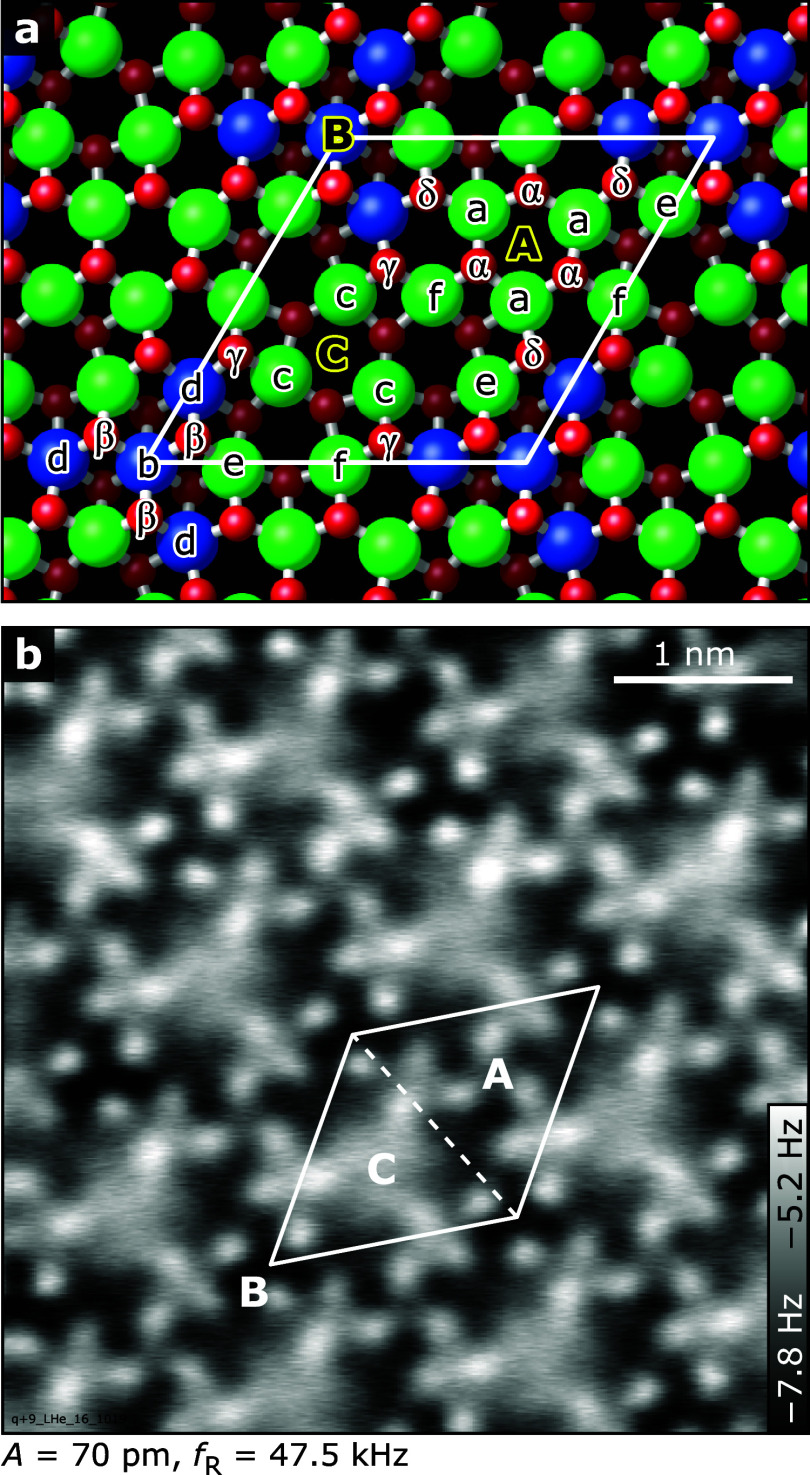
In_2_O_3_(111) surface. (a) Atomic structure including the labels used here to address individual In and O atoms (blue: 6-fold coordinated In, green: 5-fold coordinated In, red: 3-fold coordinated surface O, dark red: 4-fold coordinated O). (b) Constant-height AFM image of the bare surface acquired with an O-terminated tip at 4.7 K; the bright features are the surface O atoms.

This work follows the surface science approach, focusing on the single-crystalline In_2_O_3_(111) surface and its interaction with methanol molecules. It combines temperature-programmed desorption (TPD), X-ray photoelectron spectroscopy (XPS), noncontact atomic force microscopy (nc-AFM), and scanning tunneling microscopy (STM) techniques with density functional theory (DFT) calculations. The coverage ranges from individual molecules to multilayer formation, which translates to 1–12 methanol molecules per surface unit cell. The results are compared to the previously reported adsorption behavior of water.^16,17^ Water exhibits three distinct desorption peaks at >300 K corresponding to three dissociated species that sequentially protonate the O­(β) ions, and the remaining water OH groups (O_W_H) adsorb in a bridging position between adjacent In­(e) and In­(f). Below 300 K, the surface is further populated by three water molecules on the In­(c) sites, followed by another three water molecules on the In­(f). Finally, additional 9 water molecules per unit cell form a small cluster above B, while region A remains water-free (hydrophobic pocket).^17^

The acidity constants p*K*_a_ of water and methanol molecules are almost equal (15.7 for water, 15.5 for methanol^18^). This leads to a quite similar chemical behavior of individual molecules concerning the strength of H bonds formed by their OH groups, the dissociation energy for transferring a proton to a surface, and the strength of the interaction of the oxygen electron lone pair with cations. Consequently, our work shows–for low coverages–that the indium oxide surface is populated by methanol molecules in a very similar fashion as previously reported for water. Dissociative and molecular adsorption, adsorption sites, energies, and structural configurations are all very comparable. As for water, the first three methanol molecules in the surface unit cell dissociate and molecular adsorption is preferred for all subsequently adsorbed MeOH, which are stable below 300 K only.

Methanol and water also show characteristic differences, however. Water is capable of forming two H bonds, whereas methanol is limited to only one H bond per molecule. On the other hand, the methyl group of the methanol molecule can add a noticeable contribution of dispersion energy. These differences in the molecule–molecule interactions result in different melting points and sublimation enthalpies (≈0.524 eV for ice^19^ and ≈0.486 eV for solid methanol,^20^ both at 145 K). On indium oxide, this leads to the observed differences in the adsorption behavior at higher coverage. For water, the stronger molecule–molecule interactions result in the formation of structures with 18 molecules per surface unit cell consisting of small pile-ups of molecules (“nanoclusters”).^17^ For methanol, the coverage before multilayer formation is significantly lower with 12 molecules per surface unit cell. Three methanol molecules adsorb above the surface O_S_H stemming from the dissociated methanol and induce a reorientation of three other MeOH to optimize the H-bond network. In both cases, water and methanol, three under-coordinated indium sites In­(5c) in the unit cell remain unoccupied (akin to “hydrophobic pockets”), due to the reduced reactivity of the undercoordinated surface oxygen at these sites,^14^ which leads to a preference for nanocluster formation.^17^

## Experimental and Theoretical Methods

The experiments were carried out in two UHV systems. The home-built TPD chamber with a base pressure 1 × 10^–10^ mbar is equipped with XPS using monochromatic Al Kα (FOCUS 500, PHOIBOS 150), a differentially pumped effusive molecular beam (MB), and a quadrupole mass spectrometer (Hiden Analytics) for quantitative desorption studies. More details about the setup can be found in ref 21. Methanol (≈10 mL, filled in a glass flask) was precleaned by five freeze–pump–thaw cycles and was checked with the mass spectrometer for impurities (≈0.5% water). The reservoir pressure of the MB was 0.4 Torr and the beam diameter was 3.1 mm at the sample surface (see Supporting Information). XPS was done in grazing emission (70° with respect to the surface normal). The 200 nm thick In_2_O_3_(111) thin film used for the TPD experiments was grown on 5 × 5 × 0.5 mm^3^ yttria-stabilized zirconia by PLD as described in ref 22. The sample was mounted on a Ta plate with six Ta stripes firmly pressing it onto the plate and a thin Pt foil underneath to improve thermal contact (see Supporting Information). After initial cleaning (see below), the surface was mostly cleaned by oxidation (700 K in 1 × 10^–6^ mbar O_2_ for 10 min and cooling in O_2_ until 450 K). This was sufficient to remove contaminations from the surface (checked with XPS) while being gentle enough to not reduce and roughen the thin film notably during the course of the experiments. First, 0.3 L of methanol were dosed with the molecular beam at a sample temperature of 100 K. The area below this TPD curve corresponding to three molecules/u.c. was used to calculate the coverages of all other TPD curves. For most of the other experiments, the sample temperature was kept more than 50 K below the desired desorption peak during the exposure and a sufficient amount of methanol was dosed (taking the decreasing sticking coefficient into account). The sample temperatures were ≈72 K for α, ≈100 K for β, ≈140 K for η, and ≈210 K for γ. For the TPD curves, the sample was placed <2 cm in front of the mass spectrometer, and its temperature was ramped with 1 K/s from 100 to 500 K, recording mass-to-charge-ratios *m*/*z* of 18 (H_2_O^+^), 28 (CO^+^), 29 (CHO^+^), 31 (CH_2_OH^+^ and CH_3_O^+^), 32 (CH_3_OH^+^, O_2_^+^), and 44 (CO_2_^+^). Our TPD spectra are based on the most intense signal of the cracking pattern, *m*/*z* = 31. The desorption energies were extracted from the TPD data by mathematical inversion of the Polanyi–Wigner equation with the assumption of first order desorption kinetics and prefactor values independent of coverage and temperature.^23^ The plot of *E*_d_ vs coverage for prefactors between 10^13^ and 10^15^ is shown in Figure 2c. The end points of the *E*_d_ intervals given in Table 1 are the maximum and minimum values found within the relevant coverage intervals and also include an order of magnitude uncertainty of the prefactor. In an ideal case, the assumed prefactor values can be verified by comparison of experimental and simulated TPD curves, as described in ref 23, and successfully demonstrated also on oxide surfaces.^24,25^ However, such a comparison requires large TPD data sets with varying initial coverages and overlapping trailing edges of the individual peaks. Acquisition of such data sets is complicated on In_2_O_3_ due to the slow carbon accumulation on the surface observed during repeated methanol TPD experiments and a progressive reduction of the thin films upon repeated preparations including sputtering and annealing. Therefore, the prefactor values used in this work were assumed based on previous work,^17^ additionally justified by the similar adsorption behavior of methanol and water.

**Figure 2:**
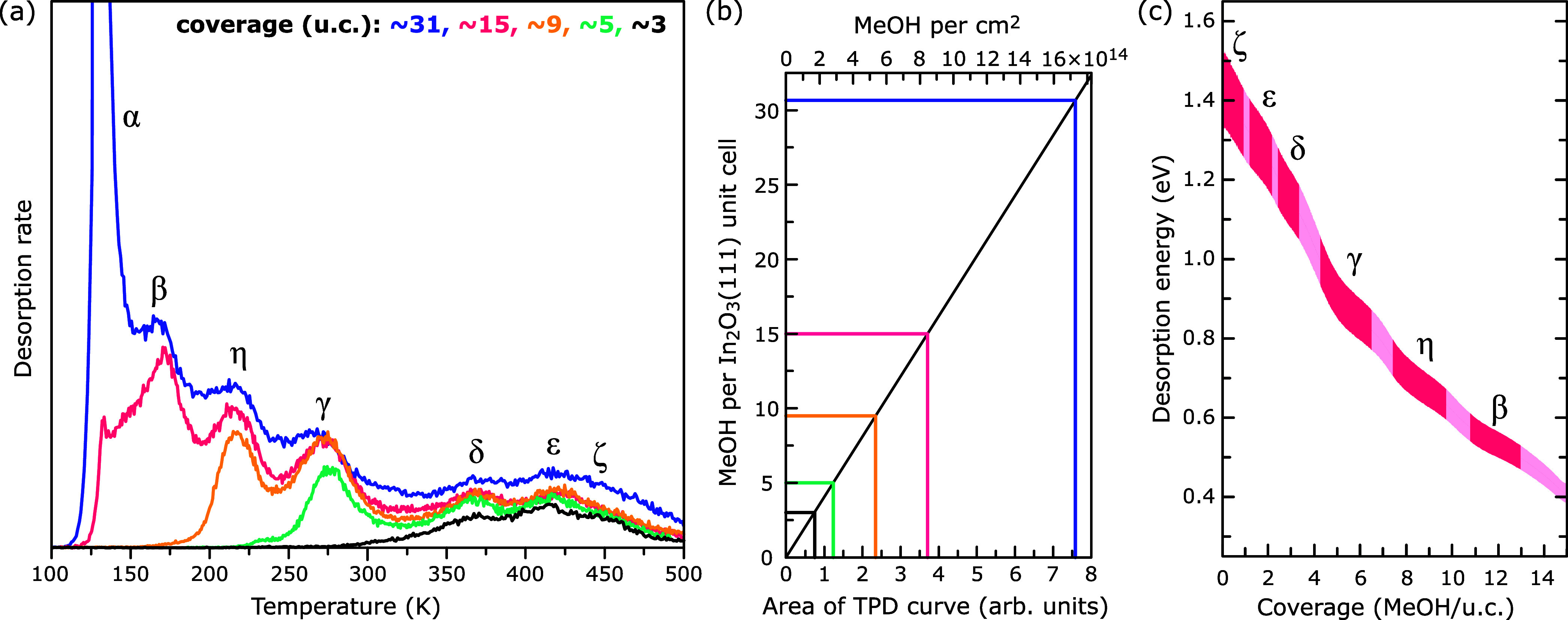
Methanol desorption from In_2_O_3_(111). (a) TPD curves of methanol (signal for *m*/*z* = 31) for ≈3, ≈5, ≈9, ≈15, and ≈31 molecules per surface unit cell (u.c.), respectively. The desorption peaks are discussed in the text. (b) Plot of the coverage using the black curve in panel (a) as reference. (c) Desorption energy versus coverage obtained from the inversion analysis of the TPD curves.

**Table 1:** Desorption Energies *E*_d_ Obtained from a Simplified Inversion Analysis (see Experimental and Theoretical Methods Section and Supporting Information) and DFT Calculations with and without Including the Grimme D3 van der Waals Corrections^31^ ^,^ ^a^

TPD peak	nominal peak coverage (MeOH/u.c.)	cumulative coverage (MeOH/u.c.)	TPD inversion *E*_d_ (eV), ν (s^–1^)	DFT + D3 *E*_b_ (eV)	DFT (w/o D3) *E*_b_ (eV)
ζ (≈450 K)	1	1	1.39 ± 0.13, 1 × 10^14 ± 1^	1.23	1.23
ε (420 K)	1	2	1.28 ± 0.12, 1 × 10^14 ± 1^	1.14	1.14
δ (370 K)	1	3	1.16 ± 0.12, 1 × 10^14 ± 1^	1.02	1.01
γ (270 K)	3	6	0.91 ± 0.14, 1 × 10^14 ± 1^	0.91	0.85
η (215 K)	3	9	0.70 ± 0.11, 1 × 10^14 ± 1^	0.71	0.62
β (168 K)	3	12	0.54 ± 0.08, 1 × 10^14 ± 1^	0.58	0.48
α (≈130 K) (see SI)	>60	>70	≈0.475 (see SI)	0.54^b^	0.40^b^

a Coverages are given as number of methanol molecules per unit cell. DFT-calculated energies *E*_b_ are differential binding energies per molecule, see Experimental and Theoretical Methods section.

b Lattice energy of solid methanol, see Supporting Information.

The second UHV system was equipped with an Omicron LT-STM/AFM operating at 4.6 K, using a differential amplifier^26^ mounted next to the scanner and qPlus sensors.^27^ Sensors with the following parameters were used: (1) resonance frequency *f*_R_ = 20.9 kHz, quality factor *Q* = 39,500; (2) *f*_R_ = 30.8 kHz, *Q* = 16,000. The noncontact AFM was used in constant-height mode with a constant oscillation amplitude of 80 pm (frequency modulation) and at a sample bias voltage of nominally 0 V. STM images were acquired at constant current, tunnelling into empty states. For the STM/AFM measurements, both an In_2_O_3_(111) single crystal and a 200 nm thick In_2_O_3_(111) thin film were used. Both were initially cleaned by cycles of sputtering (normal incidence, 10 min, 1 kV, 5 mA emission, ≈3.8 μA/cm^2^ sample current) and annealing (700 K, 10 min in 1 × 10^–6^ mbar O_2_ and cooling in O_2_ until 450 K). Cleaning between experiments was reduced to 2 min of sputtering and 2 min of oxidation. Approximately 2 mL of methanol was attached to the chamber in a glass flask and cleaned by four freeze–pump–thaw cycles. Methanol vapor was dosed from a leak valve onto the sample by backfilling the chamber with ≈5 × 10^–9^ mbar. For the AFM measurements, the structures above 300 K were prepared by dosing 2 L of methanol (5 × 10^–9^ mbar methanol for ≈9 min) on the clean In_2_O_3_(111) surface at 300 K. After the exposure, the sample was gently heated to desorb some of the molecules. Methanol does not always desorb completely, some dark features remained on the surface (see the isolated dark species in Figure 4a,b). The structures that are stable only below 300 K were prepared by dosing ≈5 × 10^–9^ mbar methanol first at room temperature for 9 min (to cover the surface with methoxy groups), followed by cooling of the sample to ≈230 K (γ), ≈170 K (η), or ≈140 K (β). For the sample transfer to the STM/AFM, the sample was further cooled to 100 K (in UHV).

DFT structure relaxations for methanol molecules on In_2_O_3_(111) were carried out with the plane-wave code PWscf of the Quantum Espresso software package,^28^ using the PBE generalized-gradient exchange-correlation functional of Perdew, Burke and Ernzerhof,^29^ Vanderbilt ultrasoft pseudopotentials,^30^ and a cutoff energy of 30 Ry for the plane-wave expansion of the wave functions. Dispersion corrections to PBE energies and forces were added by the Grimme D3 scheme.^31^ Included was only the molecule–molecule interaction between the methoxy groups; the interaction of the methanol OH groups was excluded (see Supporting Information for more details). The methanol binding energies in Table 1 are calculated as differential energies with respect to the next lower coverage as *E*_b_ = [*E*_slab_(*N*_i_) + (*N*_f_ – *N*_i_)*E*_mol_^MeOH^ – *E*_slab_(*N*_f_)]/(*N*_f_ – *N*_i_). Here, *E*_slab_(*N*_i_) and *E*_slab_(*N*_f_) are the total energies of the slabs with the initial and final number *N*_i_ and *N*_f_ of methanol molecules, respectively, and *E*_mol_^MeOH^ is the total energy of the methanol gas-phase molecule. The binding energies are reported without corrections for zero-point vibrational energies (ZPVE) and finite-temperature contributions. For comparison with previous results for the adsorption of water on In_2_O_3_(111)^16,17^ binding energies are also listed without the dispersion contribution.

The In_2_O_3_(111) surface was represented by a periodically repeated slab with a thickness of four O_12_–In_16_–O_12_ trilayers and a primitive (1 × 1) surface unit cell (160 atoms). The thickness was increased to five trilayers in the calculations of the core-level shifts (CLS). The PBE-optimized bulk lattice constant of 10.276 Å was used for the lateral slab dimensions. The atoms in the two bottom trilayers of the slab were kept frozen in their bulk positions and only the upper layers and the adsorbed methanol molecules were allowed to relax. The force convergence threshold was set to 5 meV/Å. A (2,2,1) Monkhorst–Pack *k*-point mesh for Brillouin zone integrations was sufficient for obtaining well-converged structures and binding energies.^16,17^

The *ab initio* molecular dynamics (AIMD) simulations were performed with the Car–Parrinello Molecular Dynamics (CPMD) code^32^ using the version with our recent code optimizations.^33^ All settings concerning the functional, pseudopotentials, plane-wave basis set, and the In_2_O_3_(111) slab were kept identical as in the PWscf geometry optimizations. A time step of 6 au (0.145 fs) was used for the integration of the equations of motion, and the fictitious electronic mass was set to 700 au. All hydrogen atoms were replaced by deuterium.

Final-state O 1s CLS in XP spectra were calculated using the ΔSCF approach, i.e., taking the difference between the total energy of the relaxed structure and a calculation with a core hole at a specific oxygen atom without changing the geometry. The core hole was introduced by creating a new oxygen pseudopotential from an atomic reference configuration in which an O 1s core electron was removed. Further technical details as well as a benchmark of this approach for a series of gas-phase molecules are reported in ref 34. Overall, the experimental shifts in the core-electron binding energies of the gas-phase molecules are reproduced with an accuracy of about 0.1 eV. When using pseudopotentials and charged supercells with periodic boundary conditions, one cannot calculate absolute values of the core-electron binding energies, only shifts relative to a reference atom in the same supercell. The O 1s CLS of the oxygen atoms in the first and second trilayer are reported relative to the averaged binding energy of the oxygen atoms in the third trilayer, which is the central layer in the 5-trilayer setup and is expected to represent the bulk environment (the variation of the core-electron binding energies between the oxygen atoms in the third trilayer is less than ±0.05 eV).

## Results and Discussion

### Temperature-Programmed Desorption

Figure 2 shows TPD curves of methanol on In_2_O_3_(111) ranging from 100–500 K for coverages from 3–31 MeOH per surface unit cell. The temperature ramp was stopped at 500 K to avoid the surface reconstruction with In adatoms.^15^ The nonzero intensity at 500 K as well as the increase of the desorption signal between 15 and 31 molecules/u.c. at temperatures above ≈200 K originates from desorbed methanol readsorbed on the sample holder and finally desorbing from there at the given temperature. While cooling the sample to 100–200 K before each exposure to methanol, water from the residual vacuum can adsorb on the sample holder and sample; it becomes visible in the *m*/*z* = 18 desorption signal (see Supporting Information). This water blocks a small fraction of the adsorption sites for methanol. Moreover, cycling methanol exposure followed by TPD acquisition eventually led to a buildup of carbon on the surface as seen in XPS. Presumably, the methanol molecule dissociates at defect sites (possibly including step edges) and does not recombine and desorb as an entity. The water coadsorption during cooling together with the contamination of the surface had an impact on the desorption peak ζ, which is not always well-defined (see, e.g., orange curve in Figure 2a). Only a selection of desorption curves with specific/relevant coverages is shown here, which were obtained after dosing ≈3 (black curve in Figure 2a), ≈5 (green), ≈9 (orange), ≈15 (red), and ≈31 (blue) methanol molecules per surface unit cell (Figure 2b).^21^ The nominal coverages per peak (in agreement with nc-AFM and DFT discussed below) as well as the cumulative coverage are given in Table 1.

In the TPD curves of Figure 2a, seven desorption peaks are observed. The peak at the lowest temperature, labeled α, represents desorption of the methanol multilayer following zero-order desorption kinetics. Leading-edge analysis of high coverages (10, 15, and 20 L, see Supporting Information) yields a multilayer desorption energy of ≈0.475 eV, which is in good agreement with the enthalpy of sublimation of methanol of ≈0.486 eV.^20^ With increasing temperature, the desorption peaks are labeled as follows (with the temperature at peak maximum given in parentheses): β (168 K), η (215 K), γ (270 K), δ (370 K), ε (420 K), and ζ (≈450 K). Assuming first-order desorption kinetics and desorption prefactor values of 1 × 10^14 ± 1^ (similar range as previously found for water on In_2_O_3_(111)^17^), the desorption energies extracted from the TPD data range from 0.54 ± 0.08 eV for the β peak to 1.39 ± 0.13 eV for the ζ peak, as summarized in Figure 2c and Table 1. Details on the TPD data analysis are provided in the Experimental and Theoretical Methods Section and the Supporting Information.

### X-ray Photoelectron Spectroscopy

The methanol coverages used in the TPD experiments were probed with XPS to learn more about the nature of the adsorbed species, see Figure 3. The analysis of the experimental XPS data is supported by DFT calculations of O 1s core-level shifts (CLS) for different methanol coverages (see Supporting Information). In addition, the computed CLS are used to construct the full O 1s core-level spectrum as outlined in a recent publication^34^ and to predict XPS spectra for specific methanol coverages (see Supporting Information).

**Figure 3:**
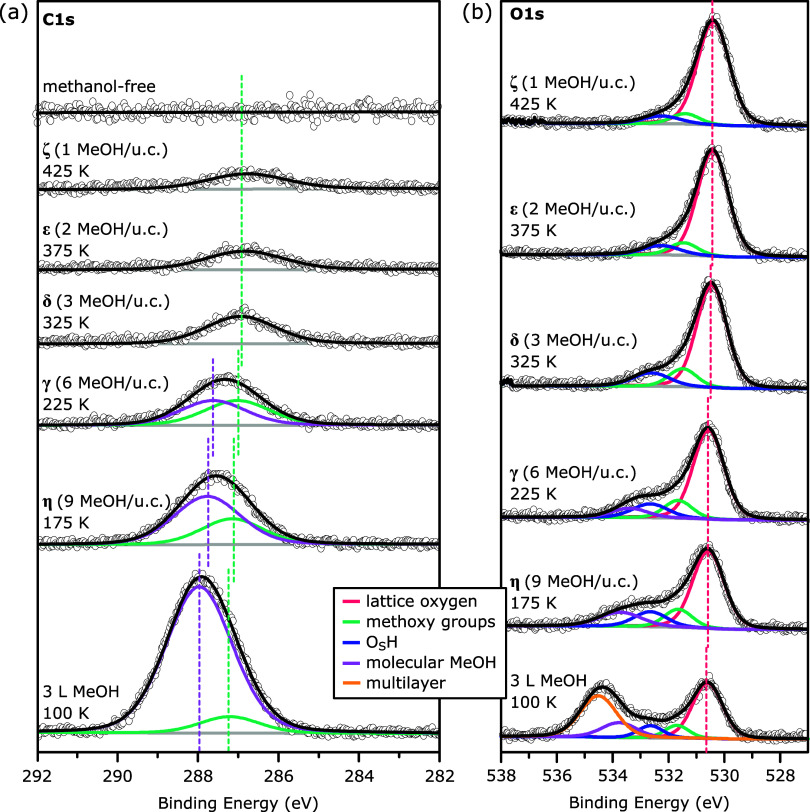
XPS of methanol adsorbed on In_2_O_3_(111). Evolution of the (a) C 1s and (b) O 1s core levels as function of the coverage on In_2_O_3_(111). The sample was first exposed to 3 L of methanol at 100 K (bottom), then heated step by step to the temperatures stated, and kept there while acquiring XPS, except for the coverages labeled ε and ζ, which were measured at 325 K. The TPD peak designations ζ– η indicate the structure/coverage still present at the surface. The spectra were obtained using monochromatic Al Kα and measured in grazing emission (70°).

In the measurements, 3 L of methanol were dosed *via* the molecular beam onto an In_2_O_3_(111) thin film at 100 K and investigated with XPS in grazing emission. Using the TPD curves as a guide, the sample was heated stepwise to specific temperatures that were kept while acquiring the spectra (except for configurations ε and ζ; here the sample was cooled to 325 K for XPS). This way, the XPS results can be related to the configurations that resulted in the respective TPD peaks. For clarity, in the following the XPS spectra are discussed in the reversed order as acquired, i.e., from low to high coverages (or high to low sample temperatures).

The first three methanol molecules (corresponding to the desorption peaks δ, ε, and ζ) adsorb dissociatively on the In_2_O_3_(111) surface, as evidenced by the position of the single-component C 1s peak at ≈286.9 eV (Figure 3a). The shoulder in the O 1s core level at ≈532.0 eV (Figure 3b) contains both, the methoxy groups and the surface O_S_H created by the adsorption of the split-off protons in a 1:1 ratio. Since the DFT calculations predict a significant difference in the O 1s core level for these two species, the shoulder was fitted with two components (green and blue in Figure 3b). The XPS peak positions are shifted to higher binding energies by 1.03 (green, methoxy) and 2.16 eV (blue, surface O_S_H) with respect to bulk oxygen (red). This is in excellent agreement with the calculated CLS of 0.96 and 2.1 eV, respectively (see Supporting Information). In comparison to the clean surface (not shown), a downward band bending of ≈0.2 eV is observed.

Increasing the coverage to 6 MeOH per unit cell (225 K, total coverage of γ) yields three methanol molecules in addition to the already-discussed three dissociated MeOH. This is reflected in both, the C 1s and O 1s core levels: In the C 1s, the doubling of the coverage leads to a shift of 0.3 eV toward higher binding energies; ≈0.1 eV of this shift is again due to downward band bending, the remaining shift combined with the broadening of the C 1s core level indicates the presence of two species on the surface. Two components of equal area and width fit this peak and correspond to molecular (287.6 eV, pink in Figure 3a) and dissociated (287.0 eV, green) methanol, respectively. In the O 1s signal, the shoulder at the high binding-energy side spreads further to even higher binding energies. Motivated again by the calculated CLS of the three different species on the surface, the spectrum is now fitted with three components, two (same as for three methanol/unit cell) corresponding to methoxy and surface OH groups (O_S_H), the third one (located at ≈533.4 eV, pink in Figure 3b) to the O in the molecularly adsorbed methanol. The ratio between molecular and dissociated species is ideally 1:2, i.e., three O atoms of the molecular MeOH versus six O atoms in the dissociated species (methoxy groups plus O_S_H). In the data shown in Figure 3b the molecular component is clearly smaller; this is due to how the experiment was conducted. The sample was kept at 225 K during XPS and methanol molecules slowly desorbed.

In the next TPD peak η (XPS at 175 K), three additional methanol molecules are present; thus, the total coverage on the surface before desorption is increased to nine MeOH (still, three of them are dissociated). In the C 1s region of the XPS spectra (Figure 3a), the molecular peak rises and the molecular and dissociated components show a ratio of ≈2:1. In the O 1s region (Figure 3b), however, the flat shoulder at the high binding-energy side spreads even more but without a clear peak structure. It already contains the methoxy groups (blue), O_S_H (green), and covalently bound MeOH (pink). Fitting this broad shoulder without more information about the species adsorbed on the surface is difficult. When keeping the components derived for configuration γ constraint to their positions, an additional component at even higher binding energy would be necessary. However, evaluation of the nc-AFM images and DFT calculations (both discussed below) show that all 9 MeOH/u.c. (three dissociated and 6 intact) of the η phase are adsorbed at the In­(5c) lattice sites In­(c), In­(e), and In­(f). This suggests that it is more natural to keep only a single molecular component in the XPS peak fit. On the other hand, in the DFT calculations of the O 1s CLS, a significant shift of the molecular components to higher binding energies is observed when going from 6 to 9 MeOH/u.c. (see Supporting Information). These observations guided the fit of the 9 MeOH/u.c. spectrum: we kept three components as in the fit for the γ phase (O_S_H, methoxy, and undissociated MeOH), but let the position of the three components freely adjust. We find that the components of the dissociated methanol molecules remain basically unchanged, but the molecular component (pink) shifts by ≈0.3 to 533.7 eV (DFT predicts a shift of about 0.5 eV) and broadens due to the fact that intact MeOH molecules occupy two different In­(5c) sites.

We could not obtain well-defined XPS data for the desorption peak β because further methanol adsorption also contributes to multilayer formation and an increase of the molecular C 1s component (bottommost spectrum in Figure 3a). In the O 1s spectrum including a large fraction of MeOH in the multilayer, a noncovalently bound molecular component (orange in Figure 3b) emerges, similar to the O 1s CLS of multilayer water.^17^ Fitting parameters and the evolution of the individual components are reported in the Supporting Information.

### Noncontact AFM and STM

Finally, scanning probe techniques were employed to elucidate the methanol overlayer structures related to the peaks observed in the TPD curves. In the constant-height AFM images, the tip–sample separation was chosen such that the most protruding species is imaged as a bright feature, i.e., on the repulsive part of the force–distance curve. Species that are located closer to the surface thus interact less repulsively (less bright) or even attractively (dark) with the tip. Note that the scanning probe experiments were performed in a different UHV chamber from the TPD and XPS investigations: the sample preparation is described in the Experimental and Theoretical Methods Section. According to TPD and XPS, the three desorption peaks above 300 K (ζ, ε, and δ) relate to the sequential adsorption (with decreasing temperature) of one, two, and three dissociated methanol molecules per In_2_O_3_(111) unit cell. Images representative of this sequential population are displayed in Figure 4. Since the lattice of the In_2_O_3_(111) surface is not visible in-between the methoxy groups, the adsorption site was determined by codosing different amounts of water at room temperature as a reference molecule with a well-known adsorption site^17^ (see Supporting Information) and confirmed by DFT calculations (see below). In the AFM images of Figure 4, single, dissociated methanol molecules (panel a, structure ζ) are identified as bright features (methoxy group), with a small, dark dot next to it (proton adsorbed on the lattice O atom). Due to the 3-fold symmetry of the In_2_O_3_(111) surface, this double feature of the dissociated MeOH is present in three orientations; one is indicated by white circles. Based on that, the methanol adsorption site is found to be the same as for water, namely the methoxy group adsorbs bridging the 5-fold coordinated atoms In­(e) and In­(f). The split-off proton adsorbs next to the methoxy group on the 3-fold coordinated O­(β) that shares a bond with the In­(e). Increasing the coverage leads to a total of two MeOH per unit cell (Figure 4b, structure ε). The second methanol adsorbs dissociatively in a site that is (initially) symmetry-equivalent to the first one. Together, the two protons on the O­(β) are imaged as an elongated dark feature (close to a corner of the unit cell). This dark feature connects the bright dots identified as methoxy groups. A pair of dissociated MeOH is indicated by white circles in Figure 4b. Finally, in Figure 4c (structure δ), all three equivalent sites around B are occupied by dissociated MeOH. The three split-off protons on the O­(β) are imaged as the dark area at the corners of the unit cell, surrounded by the three methoxy groups in In­(e)–In­(f) bridging positions.

**Figure 4:**
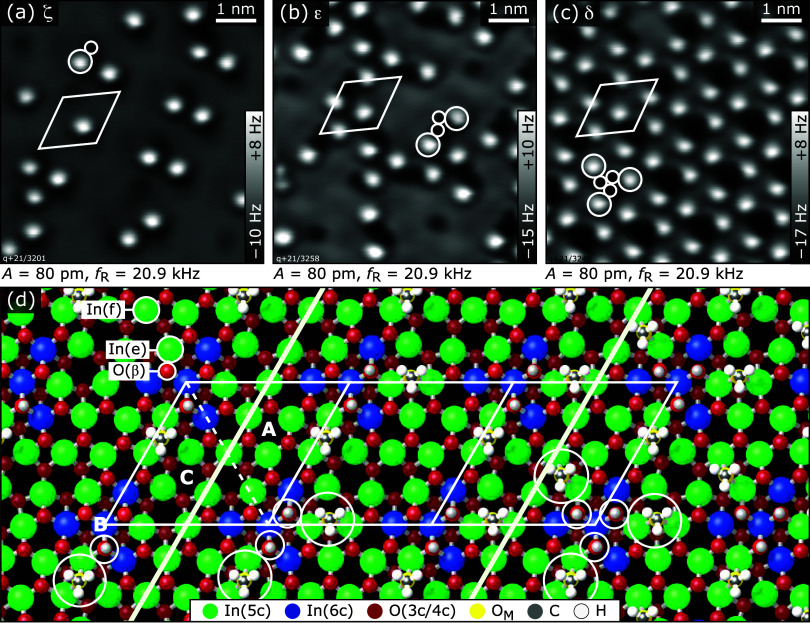
AFM images of configurations ζ– δ, formed by 1–3 molecules per In_2_O_3_(111) unit cell, respectively. The (1 × 1) substrate unit cell is drawn with solid white lines with corners located at the 3-fold rotation axis B. (a) Single, dissociated MeOH. The bright features are the methoxy groups, the dark dots next to them are the protonated surface O­(β). (b) Average coverage of two dissociated MeOH per unit cell. (c) Three MeOH per unit cell occupying symmetry-equivalent sites around the corner of the unit cell (B). The protons (dark) are surrounded by three methoxy groups (bright). (d) DFT-optimized structures for 1–3 dissociated methanol molecules per unit cell.

Adsorbing methanol below 300 K leads to molecular species on the In_2_O_3_(111) surface according to XPS. The first structure (desorption peak γ) is expected to accommodate three MeOH together with the three dissociated molecules, i.e., a total of six MeOH per unit cell. Figure 5a presents an AFM image of this coverage. The location of molecules within the unit cell was determined by comparing AFM and STM images acquired at intermediate coverages. Note that the AFM images of Figures 4 and 5 were taken on different samples, hence the lattice is rotated differently with respect to the scan directions (but not mirrored). The species protruding furthest from the surface (yellow circles in Figure 5a) are identified as the molecular MeOH adsorbed on In­(c) atoms (see also the DFT-optimized structure in Figure 5d). These MeOH are surrounded by the three methoxy groups of Figure 4c (white circles). The surface OH created by the protons (black circles) are not visible any more at this tip–sample separation. According to DFT calculations, the methoxy groups are no longer centered at the In­(e)–In­(f) bridge but move toward In­(e); see the discussion below.

**Figure 5:**
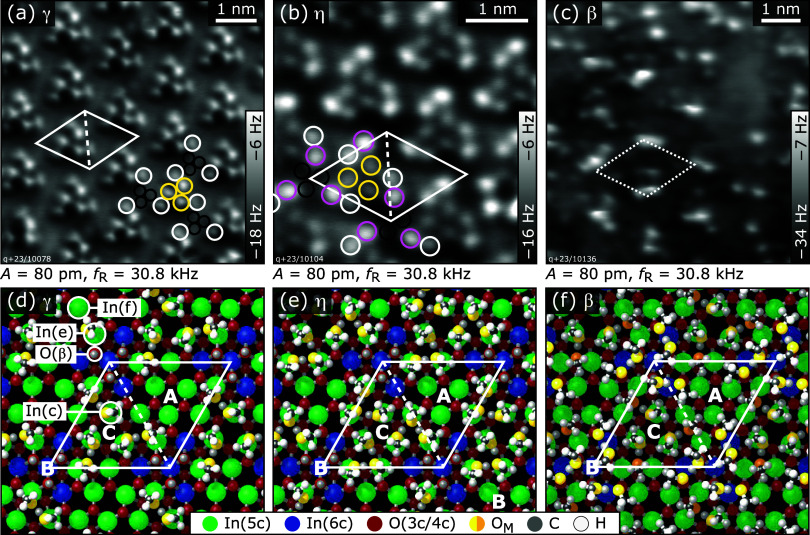
Methanol structures on In_2_O_3_(111) formed by 6–12 molecules per unit cell, corresponding to the coverages of the structures γ–β. The (1 × 1) substrate unit cell with corners located in B sites is drawn with solid white lines; the dashed line is placed to guide the eye. (a) Six MeOH per unit cell (three of them dissociated as in Figure 4c). The most protruding species are the molecular MeOH (yellow circles), framed by the methoxy groups (white circles). The protons are not visible due to the larger tip–surface separation (black circles). (b) Nine MeOH per unit cell, i.e., the previous coverage plus three additional MeOH molecules (pink circles). The central three dots are the MeOH of the previous coverage (yellow), the surrounding three bright pairs are formed by the methoxy group (white) and one additional MeOH (pink). (c) Approximately 12 MeOH per unit cell, i.e., the previous coverage plus ≈3 MeOH. (d–f) DFT-optimized structures for 6, 9, and 12 MeOH molecules per unit cell, respectively.

Continuing with the next desorption peak (structure η), three more MeOH molecules per unit cell are added. This gives a total of three dissociated and six molecular MeOH per unit cell. In the AFM image, Figure 5b, the three MeOH of the previous structure identified in the center of the arrangement (yellow circles) are surrounded by three pairs of protrusions, each located on-top of an In­(e)–In­(f) pair. According to the DFT calculations in Figure 5e, the methoxy groups moved from their positions close to In­(e) to an on-top position on In­(f) (white circles in panel b), and the three additional MeOH molecules (pink circles, now the brightest species in AFM) adsorb on-top of the In­(e) atom close to the position where the methoxy was previously attached. The last structure, before multilayer growth sets in, is phase β with a total of 12 MeOH per unit cell, i.e., three more molecules compared to the previous structure η. The AFM image of this coverage in Figure 5c shows various features in an (1 × 1) arrangement (indicated by the dashed unit cell). It was not possible to prepare a well-defined coverage for the β phase (see Experimental and Theoretical Methods Section) due to the overlap of the desorption feature with the multilayer desorption peak.

### DFT Calculations

The search for the coverage-dependent structures of methanol on the In_2_O_3_(111) surface followed the same strategy as for water in the previous studies.^16,17^ First, the preferred adsorption of a single methanol molecule in the primitive (1 × 1) surface unit cell was determined, considering both molecular and dissociative configurations. As for water, intact methanol molecules coordinate *via* their oxygen atom, O_M_, to the unsaturated In­(5c) sites. Upon dissociative adsorption, the proton converts an O­(3c) surface oxygen, O_S_, to a hydroxyl (O_S_H) and the remaining methoxy group takes either an on-top or a bridging position at the In­(5c) sites. The (1 × 1) unit cell of the In_2_O_3_(111) surface contains four nonequivalent 5-fold coordinated In­(5c) and four nonequivalent 3-fold coordinated O­(3c) sites, see Figure 1. DFT calculations were performed for all nonequivalent sites, probing both molecular and dissociative adsorption (see Supporting Information). Dissociative adsorption is clearly preferred, with a binding energy of 1.23 eV compared to 0.75 eV for molecular adsorption. In the most stable configuration, methanol adopts the same surface sites as water: the methoxy group is in a bridging position between an In­(e) and In­(f), and the proton adsorbs on the O­(β) next to the In­(e) (see Figure 4d). The preference of the O­(β) sites for the proton is an electronic effect, as shown by a detailed analyses in refs 14,16 .

Subsequently, a second and a third methanol were added to the (1 × 1) surface unit cell, again probing molecular and dissociative adsorption at all nonequivalent surface sites (see Supporting Information). In the most favorable configurations, these additional methanol molecules are also dissociated. They occupy the two sites that are symmetry-equivalent to the adsorption site of the first methanol molecule, as observed in the AFM images in Figure 4b,c. The binding energy of the added molecules slightly decreases from the first to the third molecule, from 1.23 *via* 1.14 to 1.02 eV (see Table 1), although all three molecules occupy symmetry-equivalent sites around B. This effect is caused by a substrate-mediated effective repulsion between the molecules due to surface rerelaxations.^16,17,35^ The relaxation of the surface atoms after cleavage of the crystal is partially lifted by the adsorption of the methanol molecules, which is contributing to their binding energy. However, because of the spatial overlap of the regions where the rerelaxations take place, the full extent of the energy gain is only available for the first adsorbate and is reduced for the second and third molecule.^16,17,35^

For higher surface coverages beyond three (dissociated) molecules per unit cell, molecular adsorption becomes more favorable (see Supporting Information). This is confirmed by the XPS measurements. The next three methanol molecules adsorb at the In­(c) ions around site C (Figure 5d, γ phase). They stabilize the methoxy groups of the first three dissociated methanol molecules by forming H bonds. The methoxy group moves out of the bridging position between In­(e) and In­(f) toward the In­(e) site. This is again the same structure as adopted by water molecules.

When more methanol molecules adsorb, however, characteristic differences evolve compared to the water structures. At a coverage of 9 molecules per unit cell (η phase for methanol, Figure 5e, and RE phase for water^17^), both methanol and water molecules still adsorb at the same sites, i.e., In­(c), In­(e), and In­(f). For methanol, however, there is only one distinct lowest-energy structure (Figure 5e, η phase). The methoxy groups move to In­(f) and receive H bonds from the two intact molecules at the In­(c) and In­(e) sites, maintaining the 3-fold symmetry of the surface. This structure is also a local energy minimum for water, but several others with similar and even lower energies coexist. Due to their ability to form two H bonds, the water molecules can start forming a 2D hydrogen-bond network by breaking the 3-fold symmetry. The energy to flip water molecules and rearrange the H-bond network is small. Therefore, several alternative structures with similar energies exist, which differ in the position of the O_W_H groups and the orientation of the intact water molecules. Some even contain a fourth dissociated water molecule.^17^ These water structures can easily transform into each other, which is why the water structure with 9 molecules per unit cell does not appear as a distinct desorption peak in TPD but as a broad ‘rearrangement’ feature (RE),^17^ in contrast to the η peak in the methanol TPD (Figure 2).

At higher coverages, the differences in the structures of methanol and water on In_2_O_3_(111) become even more pronounced. For water, the binding energy of molecules at the In­(a) site is always smaller than the lattice energy of ice (a representative quantity to characterize the molecule–molecule interactions). This is why water preferably forms small clusters with about 9 molecules above the B site (total water coverage of 18 molecules per unit cell) while the In­(a) sites remain empty, forming ‘hydrophobic pockets’.^17^ In the water clusters, the average binding energy per molecule is even slightly larger than the lattice energy of ice. The size of about 9 molecules is determined by an optimal saturation of the two H bonds that can be formed by each water molecule.

Solid methanol, on the other hand, has a smaller lattice energy than ice. Since the next higher coverage after the η phase contains 12 MeOH molecules, one might speculate that methanol now occupies the three In­(a) sites to form a full first layer before a second layer starts forming. However, despite intensive search, in the best structure with 12 MeOH, the binding energy of methanol molecules on In­(a) was only 0.40 eV. This is clearly less than the calculated lattice energy of solid methanol of 0.542 eV (the experimental lattice energy derived from the sublimation enthalpy is 0.549 eV, see Supporting Information for a discussion of lattice energy vs. sublimation enthalpy).

To search for alternative structures with lower energy, *ab initio* molecular dynamics (AIMD) simulations and a simulated annealing approach were applied. Many initial configurations of differently distributed dissociated and undissociated methanol molecules were used as starting points. The structures were equilibrated at 360 K for 20 ps and then quenched to zero temperature in about 50 ps. With this procedure we identified one unique structure that was significantly lower in energy than all the others by about 0.25 eV per unit cell compared to the second-best configuration. In the best structure (see Figure 5f), the 3 additional MeOH are adsorbed on-top of the surface O_S_H groups that originated from the three dissociated molecules, thereby receiving an H bond. Each of the three molecules also forms an H bond to a MeOH molecule that was initially sitting at the In­(e) site. These H-bond receiving molecules, however, are pulled upward and interact no longer with In­(e). Instead, they form a new H bond to a surface O­(δ). Lifting the MeOH molecules from the In­(e) site allows the methoxy groups of the dissociated molecules to move back from the In­(f) on-top position to the more favored In­(e,f) bridge site (the same as taken in the low coverage regime with only 3 molecules per unit cell). Altogether, this leads to a binding energy of the last three MeOH molecules of 0.58 eV, outweighing the lattice energy of solid methanol. The top three MeOH slightly break the 3-fold symmetry at site B. However, if the 3-fold symmetry is enforced in the DFT relaxation, the energy increases by only 0.04 eV per unit cell.

The proposed structure of the β phase can be correlated with the AFM image in Figure 5c with its periodic protrusions that also break 3-fold symmetry. An interesting feature of the β structure is that the MeOH on-top of O_S_H and the former In­(e) MeOH form three pairs around site B (MeOH with bright yellow oxygen in Figure 5f) that are not coupled to the other MeOH molecules on the surface. Therefore, if the on-top MeOH of one of the pairs is removed and the connected MeOH flips back to its previous In­(e) position, the other adsorbates are hardly perturbed. DFT calculations confirm that the binding energy of the on-top MeOH is independent whether one, two, or all three pairs are present at site B (i.e., the binding energy of the last MeOH is about the same for structures with 10, 11, or 12 molecules in the unit cell, see Supporting Information). The loss of one or even two on-top MeOH at B site would explain why the motifs in the AFM image of Figure 5c are not uniform, despite showing the (1 × 1) periodicity.

## Conclusion

This study explores the interaction of methanol, the desired product of CO_2_ reduction, with the In_2_O_3_(111) surface at various coverages in UHV. The findings demonstrate very good agreement between experimental observations and DFT calculations. Both water and methanol exhibit similar chemical behavior due to their comparable acidity constants (p*K*_a_ of 15.7 for water and 15.5 for methanol), affecting their adsorption on the indium oxide surface. They show similar adsorption characteristics, namely dissociation at low coverages up to three molecules/u.c., and adsorption as molecular species at higher coverages that are unstable above 300 K. However, methanol and water differ in their hydrogen bonding capabilities. Water can form two hydrogen bonds, methanol only one. This distinction influences their adsorption behaviors at the highest coverage considered in this work (prior to multilayer adsorption). Water forms nanoclusters made of 9 water molecules (at a total coverage of 18 H_2_O/u.c.) at one specific location of the surface unit cell and leaves one-half of the unit cell empty (hydrophobic pocket). Methanol, however, exhibits a lower maximum coverage of only 12 molecules per surface unit cell, where only three of them arranged into small clusters on-top of the protonated surface O­(β). Similar to water, one-half of the unit cell remains methanol-free. In conclusion, this work provides a firm basis for the understanding of the In_2_O_3_–MeOH interaction on the atomic level, a first step toward a full description of the chemistry of the various C_1_ species on the surface.

## Supplementary Material



## Notes

Additional Experimental Data: TPD: Multilayer desorption; TPD: Water coadsorption from the residual vacuum; STM and AFM: Methanol structures; STM and AFM: Adsorption site determination; STM and AFM: Mixed methanol coverages; XPS: Survey of the In_2_O_3_(111) surface; XPS: Peak fitting parameters; calculation of O 1s core level shifts; prediction of XPS spectra from computed CLS. Additional DFT Results: Solid methanol reference calculations; atomic structure of the adsorbate-free surface; adsorption of single methanol molecules; adsorption of methanol pairs; adsorption of methanol trimers; structures with 12 adsorbed methanol molecules (β phase); surface phase diagram (PDF)
