# Supplementary material for: Revealing the Intricate Structure of Surface Phases of Methanol on In2O3(111)
Source: J Phys Chem C Nanomater Interfaces. 2026 Jan 28;130(6):2341–51. doi: 10.1021/acs.jpcc.5c07043 (PMC12908149; doi:10.1021/acs.jpcc.5c07043)
Supplement: Supplementary file 1 [file jp5c07043_si_001.pdf]

Supporting Information

Revealing the Intricate Structure of Surface Phases of Methanol on $In_2O_3(111)$

Andreas Ziegler[1], Chiara I. Wagner[2], Hao Chen[2], Matthias A. Blatnik[2,3], Alexander Wolfram[4], Anne Brandmeier[1], Zdeněk Jakub[3], Michele Riva[2], Jiri Pavelec[2], Michael Schmid[2], Ulrike Diebold[2], Bernd Meyer[1], Margareta Wagner[2,*]

[1]Interdisciplinary Center for Molecular Materials (ICMM) and Computer Chemistry Center (CCC), Friedrich-Alexander-Universität Erlangen-Nürnberg (FAU), 91052 Erlangen, Germany

[2]Institute of Applied Physics, TU Wien, 1040 Vienna, Austria

[3]Central European Institute of Technology (CEITEC), Brno University of Technology, 61200 Brno, Czech Republic

[4]Lehrstuhl für Physikalische Chemie II, Friedrich-Alexander-Universität Erlangen-Nürnberg (FAU), 91058 Erlangen, Germany

Email: wagner@iap.tuwien.ac.at
bernd.meyer@chemie.uni-erlangen.de

Supporting Information:

Additional Experimental Data

1) TPD: Multilayer desorption S3

2) TPD: Water co-adsorption from the residual vacuum S4

3) STM & AFM: Methanol structures S5

4) STM & AFM: Adsorption site determination S7

5) STM & AFM: Mixed methanol coverages S8

6) XPS: Survey of the $In_2O_3(111)$ surface S10

7) XPS: Peak fitting parameters S11

8) Calculation of O1s core level shifts S12

9) Prediction of XPS spectra from computed CLS S13

Additional DFT Results

10) Solid methanol reference calculations S15

11) Atomic structure of the adsorbate-free surface S17

12) Adsorption of single methanol molecules S19

13) Adsorption of methanol pairs S22

14) Adsorption of methanol trimers S24

15) Structures with 12 adsorbed methanol molecules (β phase) S26

16) Surface phase diagram S29

Additional Experimental Data

1) TPD: multilayer desorption

The multilayer desorption of methanol was investigated from desorption experiments of nominal 10, 15 and 20 L, respectively, corresponding to approximately 80, 120, and 150 molecules per surface unit cell. Analysis of the leading edge of the desorption curves yields a desorption energy of ≈0.47 eV. The progression of the multilayer desorption peak across 2–20 L is illustrated in Figure S1.

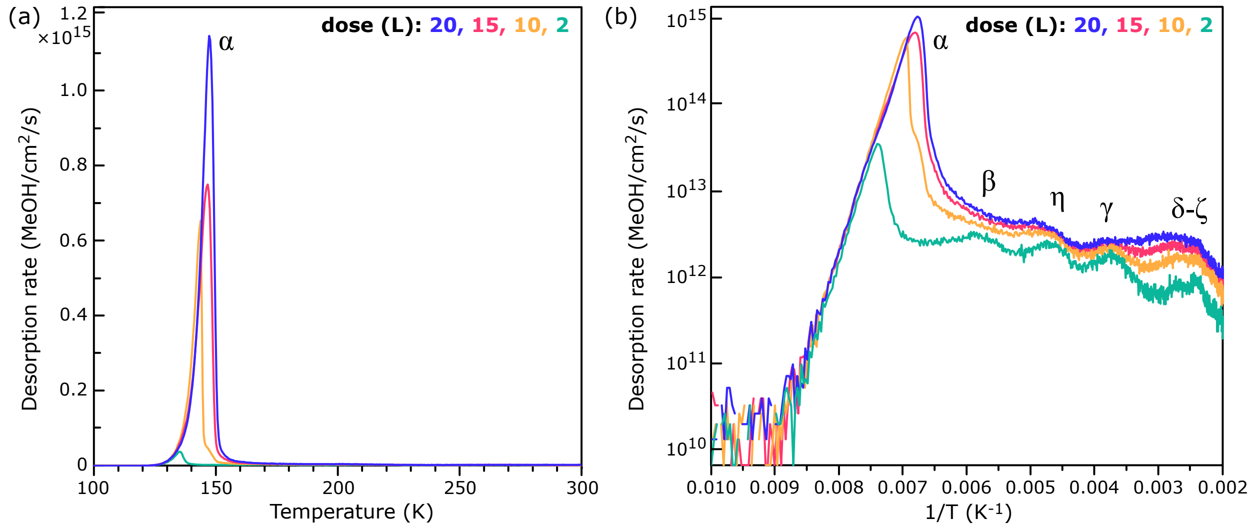

Figure S1. Multilayer desorption curves of methanol from $In_2O_3(111)$, showing the leading edge of the zero-order desorption. The curves are shown in (a) linear scale over T, and (b) logarithmic scale over $1/T$. Heating rate: $1\,Ks^{-1}$; $m/z = 31$.

2) TPD: Water co-adsorption from the residual vacuum

For the TPD experiments, specific amounts of methanol were dosed onto the $In_2O_3(111)$ surface via the molecular beam at 100–200 K sample temperature. The dosing (exposure) temperature was chosen such that the methanol desorption features of a specific coverage were at least 50–100 K higher to maintain a high sticking coefficient. While cooling the sample (≈15 min from 520 to 100 K), water from the residual gas is able to adsorb on the sample holder and areas of the sample not saturated by methanol. Figure S2 displays desorption curves of two TPD experiments, where ≈3 and ≈15 molecules/unit cell were dosed, respectively, and the desorption of both methanol and water was monitored. The water desorption curves (blue) show a prominent broad desorption feature at around 300 K, which is attributed to water desorption from the tantalum sample holder (see inset in Figure S2). The less intense peaks at around 450 K are most likely related to water desorbing from $In_2O_3(111)$, see Ref. [3].

The inset in Figure S2a shows the sample with a thin platinum foil underneath mounted onto the tantalum sample holder. The size of the molecular beam can be seen as bluish circle in the center of the sample after adsorbing a thick CO_2 multilayer ice film.

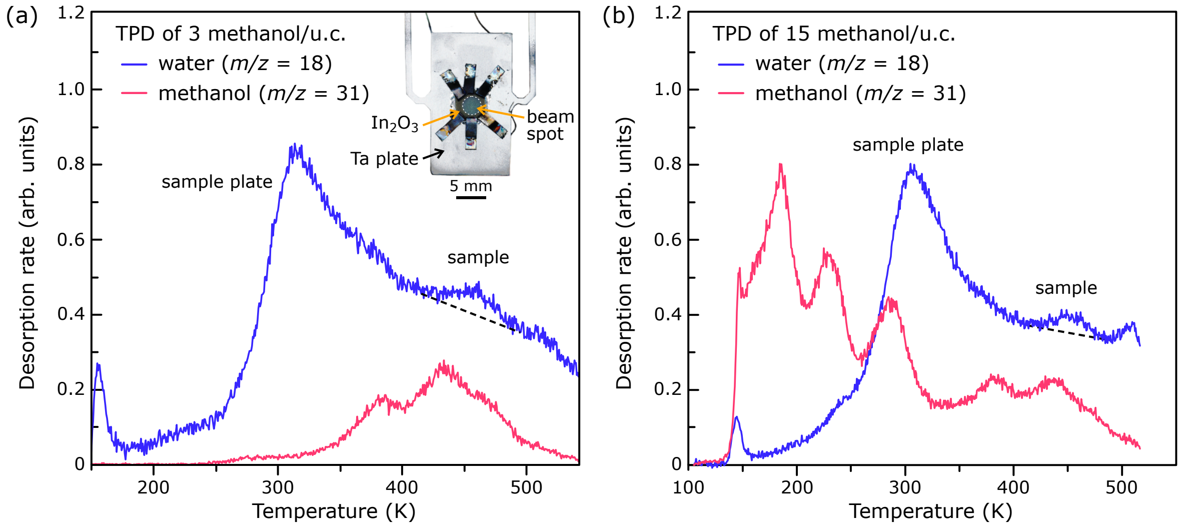

Figure S2. Water adsorption on the sample holder and sample during the TPD experiments. Desorption curves of (a) ≈3 and (b) ≈15 methanol per unit cell, tracing methanol (m/z = 31, red curves) and water (m/z = 18). Note that in (a) the sample was cooled to 150 K and in (b) to 100 K prior to methanol exposure, *i.e.*, the cooling in (b) took ≈5 min longer.

3) STM & AFM: Methanol structures

Figure S3 illustrates AFM and STM images of various methanol structures on $In_2O_3(111)$. At low coverages of 3–6 methanol molecules per surface unit cell (denoted as γ and δ, respectively; panels a–c and d–f), the empty-state STM images are dominated by bright triangular (δ) or round (γ) features associated with protons adsorbed around B. Methoxy groups and methanol molecules appear as dark dots (δ) or dark regions (γ) positioned between these features.

When the coverage increases to 9 methanol molecules per unit cell (η; Figure S3g–i), AFM reveals a well-ordered structure. STM images at this coverage display a range of appearances, from seemingly disordered features (panel h and the large image in panel i; both acquired with the same tip as in panel g) to well-ordered features (inset with black frame in panel i). Despite these differences, both types of images correspond to the same underlying structure and features observed in AFM. We assume that defects in the methanol layer close to the surface and between the regular methanol arrangements may cause different electronic properties influencing the STM images, while the main features imaged in AFM still arrange in the same pattern.

At a coverage of 12 methanol molecules per unit cell (structure β; Figure S3j–l), the proximity (in temperature) to the multilayer desorption peak makes it difficult to prepare a well-defined surface. The images show a mixture of features with (1×1) periodicity, but no clear repeating unit can be discerned.

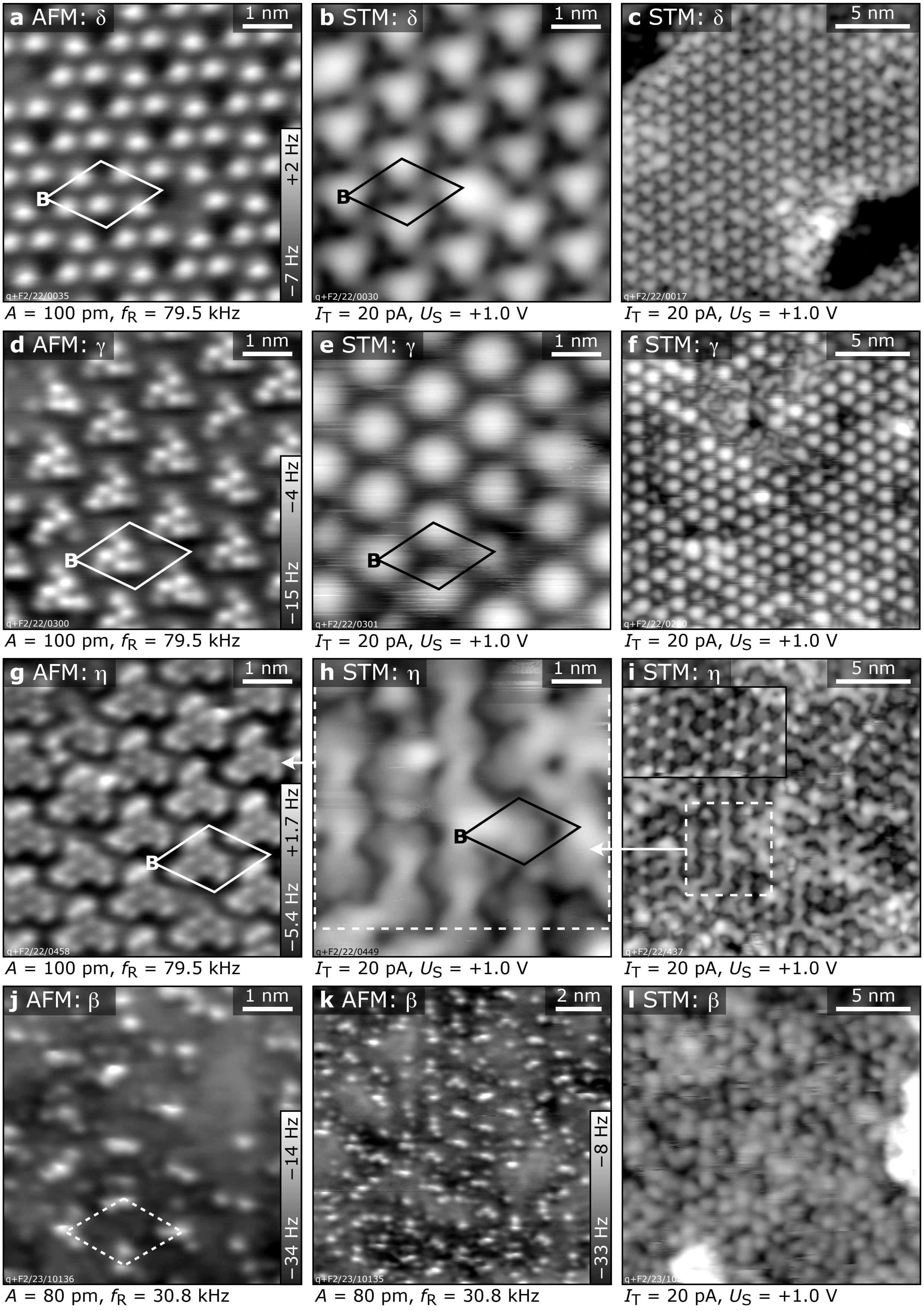

Figure S3. Comparison of the different methanol structures as imaged by AFM and STM.

4) STM & AFM: Adsorption site determination

According to XPS analysis, both methanol and water dissociate upon adsorption on $In_2O_3(111)$ at room temperature. The adsorption behaviour of water on this surface is well-characterized and discussed in Refs. [1–3]. Specifically, the proton from water adsorbs at an O(β) site, while the O_WH group bridges between In(e,f) sites adjacent to it. The three-fold symmetry of the $In_2O_3(111)$ surface provides three equivalent adsorption sites arranged around the high-symmetry axis B.

Using water as a reference, we identified the adsorption site of dissociated methanol by sequentially dosing small amounts of methanol and water onto the $In_2O_3(111)$ surface at room temperature. Figure S4 presents both STM and AFM images of such a surface. In the STM image (panel a), OH groups (from water dissociation) appear as bright triangular features positioned at the corners of the unit cell in B, while each methoxy group appears as a small dark dot. In the AFM images (panels b and c), O_WH groups (indicated by white circles encompassing 3 dissociated water molecules each) exhibit lower contrast due to their smaller geometric height, whereas methoxy groups protrude further from the surface into the vacuum, resulting in brighter features in the repulsive imaging regime. Our findings, supported by DFT calculations, indicate that methoxy groups adsorb at the same sites as the O_WH groups. As shown in Figure S4, the three sites surrounding B are occupied by a mixture of these adsorbates.

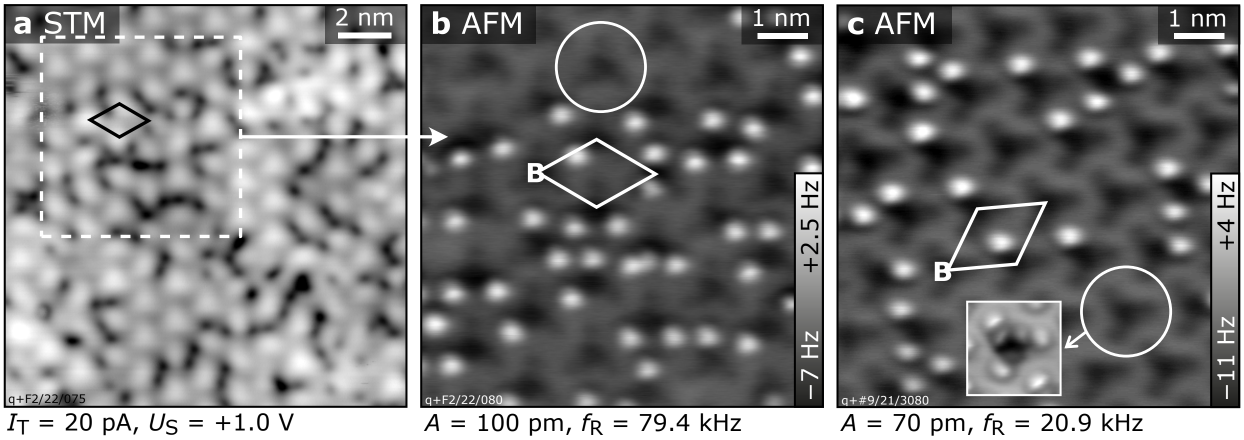

Figure S4. Methanol and water on $In_2O_3(111)$. (a) STM image of a mixture of adsorbed methanol and water molecules. (b) AFM image of the same area as in (a), and (c) AFM image of a different experiment. The inset in (c) shows three dissociated water molecules around B imaged at smaller tip–sample separation.

5) STM & AFM: Mixed methanol coverages

The adsorption sites of the methoxy groups evaluated above serve as references for experimentally determining the locations of molecular species at higher methanol coverages. To this end, a surface with a methanol coverage varying between 3 and 6 molecules per unit cell was prepared. In the STM image (Figure S5a), individual methoxy groups are again identified as small, dark dots (indicated by white arrows), see also Figure S3b. Regions with higher local coverages exhibit interactions with the STM tip, manifested as horizontal lines, and less distinct dark features. Despite this, the overall pattern of bright features associated with the three O_SH groups around area B is discernible. When the same grid is overlaid onto the corresponding AFM image (Figure S5b), it reveals that the molecular species (marked by yellow circles) are confined to one half of the unit cell (labeled C). Using the reference grid to align with the atomic structure of the surface enables the identification of these species' positions on-top of the three In(c) atoms surrounding site C. The methoxy groups observed in the STM image can also be located in the AFM image, although they are hardly visible, appearing with nearly the same frequency shift (grey level) as the surrounding surface.

Once the adsorption sites of the three methanol molecules are identified, they can serve as reference points at even higher coverages. Figures S5c–e display AFM images captured at progressively reduced tip–sample distances, covering a methanol coverage range from 6 to 9 molecules per unit cell. The motif corresponding to 6 methanol molecules is readily recognizable (several occurrences in the left half of panel d), with white and yellow circles indicating methoxy groups and methanol molecules, respectively. Additionally, the motifs formed by 7, 8, and 9 methanol molecules per unit cell are discernible.

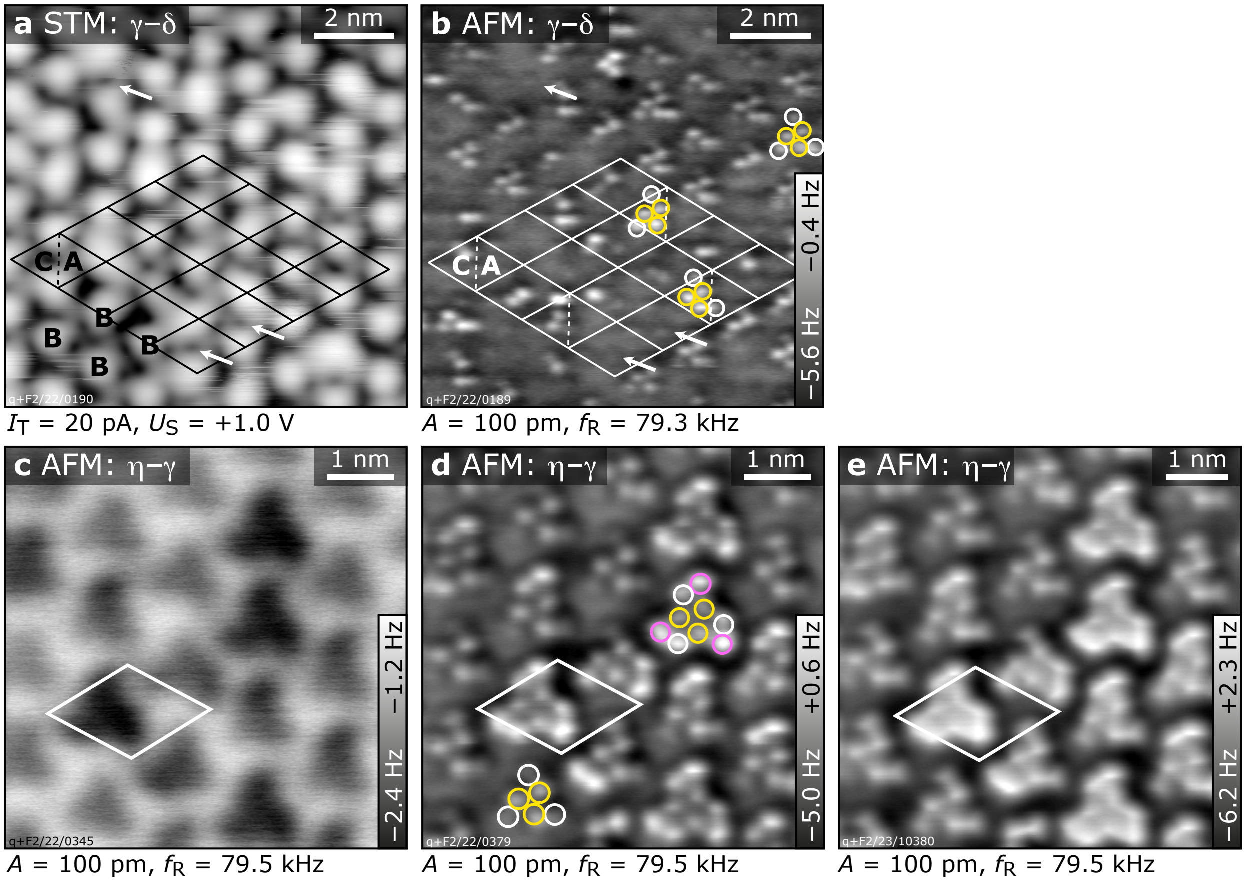

Figure S5. Mixed coverages of methanol on $In_2O_3(111)$. (a,b) Surface prepared with 3–6 methanol species per unit cell (corresponding to the range between the TPD peaks δ and γ) imaged with AFM and STM. (c–e) Surface prepared with 6–9 methanol species per unit cell (corresponding to the range between the TPD peaks γ and η) imaged with AFM. The tip–sample distance decreases from panel (c) to (e).

6) XPS: Survey of the $In_2O_3(111)$ surface

Figure S6 shows an overview photoemission spectrum of the clean $In_2O_3(111)$ surface as prepared for the methanol TPD experiments (thin film sample). The position of the sample is optimized to avoid signals originating from the clips (made of nicrofer) holding the sample on the plate (tantalum). Also avoided are bare YSZ patches at the edges of the $5 \times 5\,mm^2$ sample that are not covered by the $In_2O_3(111)$ thin film due to the sample mounting during thin film growth. The absence of carbon impurities is shown in Figure 3 (main text).

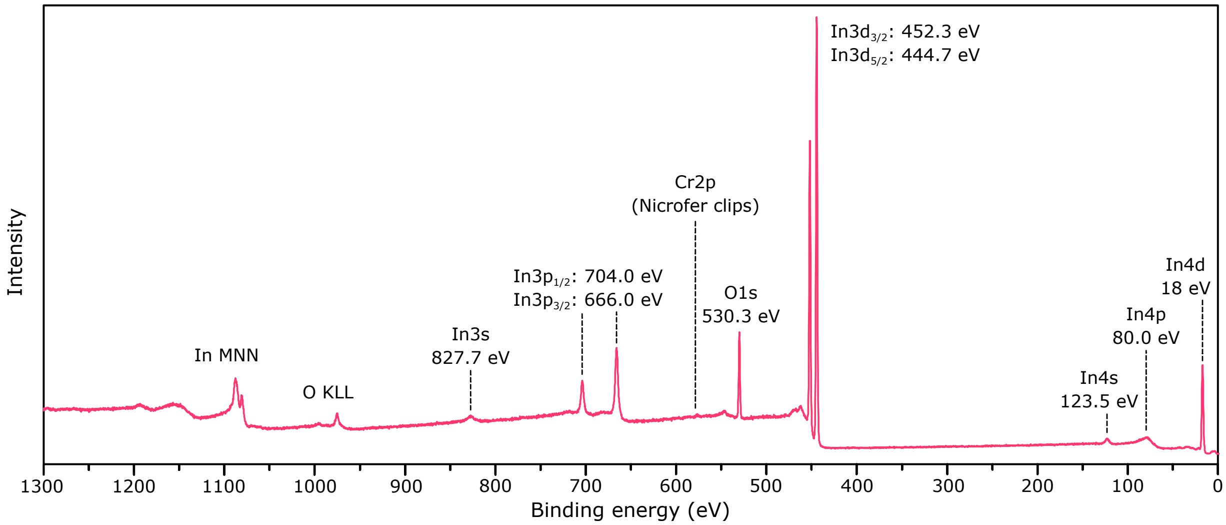

Figure S6. XPS survey spectrum of the bare $In_2O_3(111)$ surface prior to the methanol TPD experiments (grazing emission, monochromatic Al K_α).

7) XPS: Peak fitting parameters

All fitting parameters of the XPS O1s core level for the fits displayed in Figure 3 of the main text are listed in Table S1.

T	TPD	O1s	FWHM	Area	O1s MeO	FWHM	Area	O1s O_SH	FWHM	Area	O1s mol	FWHM	Area	O1s mol	FWHM	Area
100	β	530.6	1.27	1392.9	531.7	1.0 |l	251.4	532.65	1.04 |B	251.4 |B	533.77	1.48 |C	436	534.51	1.53	1252.4
175	η	530.57 |u	1.333	2148.4	531.65 |l	1.17 |u	437.5 |l	532.65	1.4	437.5 |B	533.72	1.78	521.3 |u			
225	γ	530.56 |l	1.285 |l	2441.3	531.66 |l	1.17 |l	437.5	532.65	1.5	437.5 |B	533.39	1.49	281.7			
325	δ	530.45 |l	1.27	2719.2	531.48	1.17 |l	440.6	532.61	1.56	440.6 |B						
375	ε	530.41	1.301	2857.5	531.46	1.17 |l	304.7	532.26 |l	1.56	304.7 |B						
425	ζ	530.4	1.35	2932.8	531.4	1.17 |l	252.9	532.25 |l	1.56	252.9 |B						

Table S1. XPS fitting parameters of the UHV experiments. The peak shape is modelled by a Gaussian/Lorentzian product formula with a weighting of G:L = 70:30. The actual XPS curves and fits are shown in the main text. Temperature T is in K, all core-electron binding energies are in eV. Constraints: l (lower bound), u (upper bound), f (value fixed), B (same as for methoxy O1s), C ($1.47 \times$ O1s of O_SH).

8) Calculation of O1s core level shifts

	clean surf	δ (325 K)	γ (225 K)	η (175 K)	intensity
Om3m				3.168	
Om2m			2.388	2.873	
Om1d		0.958	1.343	1.896	
O1ta	-0.170	-0.252	-0.064	-0.129	0.932
O1tb	-0.877	2.091$^{(*)}$	2.096$^{(*)}$	2.243$^{(*)}$	1.0
O1tc	-0.118	-0.114	-0.202	-0.159	0.921
O1td	-0.549	-0.249	-0.243	-0.196	0.945
O1ba	0.125	0.118	-0.060	-0.010	0.795
O1bb	-0.099	-0.259	-0.036	-0.007	0.767
O1bc	-0.214	0.071	0.012	0.104	0.732
O1bd	-0.375	0.161	0.229	0.415	0.703
O2ta	0.026	-0.102	0.036	0.083	0.608
O2tb	0.172	-0.183	0.042	-0.049	0.580
O2tc	-0.252	0.089	0.081	0.128	0.565
O2td	0.277	0.216	0.065	0.055	0.556
O2ba	-0.073	0.111	0.081	0.104	0.466
O2bb	0.068	0.024	0.017	0.052	0.462
O2bc	-0.049	-0.031	0.040	0.009	0.447
O2bd	0.003	0.058	0.040	0.036	0.429
bulk	0.0	0.0	0.0	0.0	6.24

$^{(*)}$ O_SH group formed by the dissociative adsorption of methanol

Table S2. List of relative core-electron binding energies (in eV) for the clean surface and the three phases with 3, 6, and 9 methanol molecules per unit cell. Additionally, the relative intensities obtained from the depth of each O atom in the structure is listed. The intensity of the bulk is calculated as the intensity of the 3rd–10th layer and adds significantly to the overall intensity. 'Om1d' refers to the oxygen atom of the dissociated methanol molecule (first methanol species adsorbing on the surface), 'Om2m' denotes the oxygen atom of the first molecularly adsorbed methanol (second methanol species), and 'Om3m' is the second molecular species on the surface (but overall the third methanol species). 'O(1/2)(t/b)(a–d)' indicates an oxygen atom in the first/second O–In–O trilayer, located above (top) or below (bottom) of the sandwiched In layer; the inequivalent O atoms within a top/bottom layer are labeled a–d.

9) Prediction of XPS spectra from computed CLS

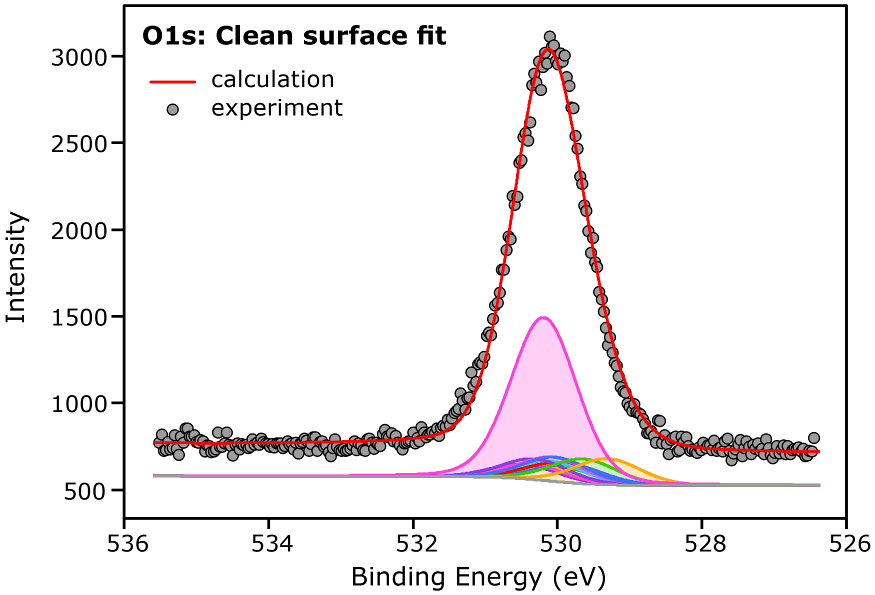

Figure S7. Fit to the experimentally-measured O1s spectrum of the clean $In_2O_3(111)$ surface. The spectrum is fitted using the calculated relative binding-energy shifts as well as the relative intensities outlined in Table S2. Using only three free fit parameters we obtain a very good fit to the clean surface spectrum and use the resulting absolute binding-energy position (530.19 eV), full width at half maximum (1.19 eV) and Gaussian-to-Lorentzian ratio (77:23) to predict O1s XPS spectra of the methanol-covered surfaces. The pink component at 530.19 eV corresponds to the bulk contribution, which appears to be very intense, because for each of the upper two layers each of the eight symmetry inequivalent oxygen atoms are considered as individual peaks.

To compare the experimental spectra with calculated core-level binding energy shifts, we constructed the full O1s core level spectrum as outlined in a recent publication.[4] Briefly, we obtain the relative binding-energy shifts as well as the depth (intensity) of each oxygen atom from DFT calculations. The deeper an oxygen atom is located within the structure, the more attenuated the photoelectrons emitted from this atom are. We estimate this attenuation using an inelastic mean-free path of 17.44 Å obtained from the TPP2M algorithm using a kinetic energy of 960 eV (O1s measured with Al K_α radiation).[5] Since we measure in 70° emission, this corresponds to an effective inelastic mean-free path (attenuation depth) of 5.95 Å. We do not get information about the peak shape of the individual contributions from our calculations. Instead, we use the O1s core level of the clean surface as our reference system. Taking the calculation results from the clean surface, we constructed a fit model with the relative binding energies and relative intensities. Since the deeper-lying oxygen atoms contribute significantly to the overall intensity, we also added an additional peak at 0 eV relative binding energy with a relative intensity obtained by summing over the intensity from the 3rd to the 10th trilayer. We obtain a very good fit to the surface in Figure S7

using only three parameters, which are the overall position (530.19 eV) and the two peak shape parameters (full width at half maximum and Gaussian–Lorenzian ratio). With this calibration, we can take any other calculation and predict the full O1s spectrum for various methanol coverages, see Figure S8. We reproduce the changes to the molecular contribution (between γ and η in Figure 3b) at around 534 eV, which decreases in intensity and shifts to lower binding energy as methanol molecules desorb from the surface. This further supports our assignment of the desorption of methanol as a mixture of different binding motives on the surface. The predicted spectra are not a perfect match to the experimental data, as especially the peak widths are too small. This was not the case for phenylphosphonic acid on rutile TiO_2(110), the benchmark system for which this method was developed,[4] but we suspect that the weaker bond between the methanol and the surface, the more flexible adsorption configuration, and inhomogeneous adsorption (partial desorption during the XPS measurement) cause a peak broadening, similar to the broad peaks we observed experimentally for water on In_2O_3(111).[3] All details for the predicted spectra are listed in Table S2.

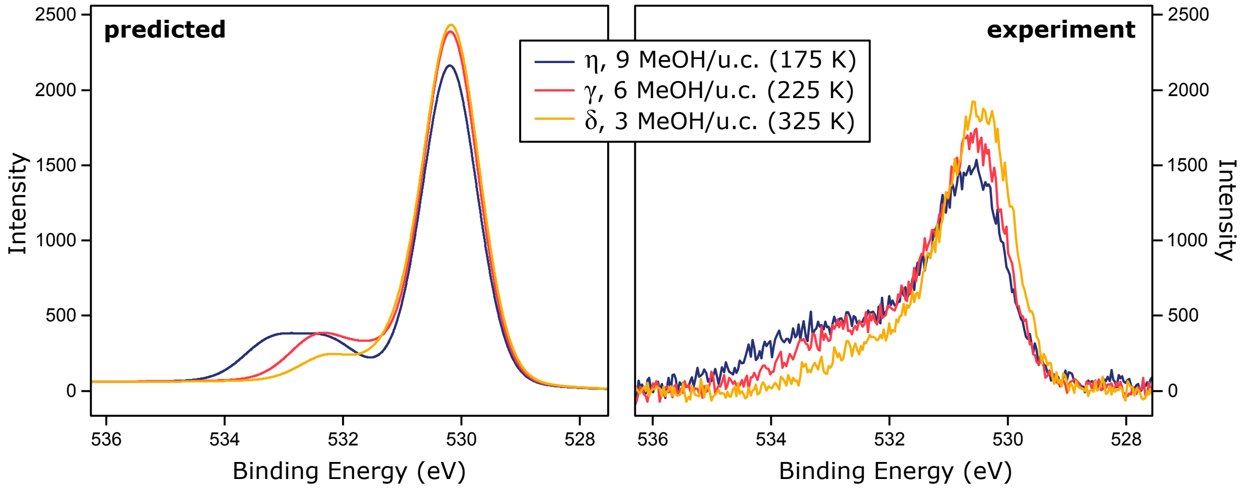

Figure S8. Comparison of predicted and measured O1s core-level spectra after annealing the methanol-covered In_2O_3(111) surface to the indicated temperatures, resulting in the loss of molecules. The predicted spectra are scaled to the peak area of the corresponding experimental spectrum. The predicted spectra reproduce the general trends observed in the high-binding energy shoulder of the experimental data, but the peak width of the individual components is too small. For a better visual comparison, a Shirley background is subtracted from the experimentally measured spectra.

Additional DFT Results

10) Solid methanol reference calculation

The reference calculations for solid methanol were done for the low-temperature ground-state α-phase.[6,7] It has an orthorhombic unit cell with space group symmetry $P2_12_12_1$ (no. 19) containing 4 methanol molecules. The experimental lattice parameters at 15 K are $a = 4.873$ Å, $b = 4.641$ Å, and $c = 8.867$ Å.[6,7] The same pseudopotentials and plane-wave cutoff as in the DFT calculations for methanol molecules on the $In_2O_3(111)$ surface together with the PBE functional and a (6,6,4) Monkhorst-Pack k-point mesh were used.

In general, PBE without dispersion corrections exhibits at least a similar, but often an even better accuracy for hydrogen bonds than most dispersion-corrected functionals (even hybrid functionals), see for example the results for the WATER27 benchmark set of small water clusters in Ref. [8]. On the other hand, dispersion corrections cannot be completely neglected for methanol because of the methyl groups. To find an optimal setup for a Grimme D3 dispersion correction,[8] we optimized the structure of solid methanol in the α-phase and calculated the lattice energy E_b for different choices of which atoms are included/excluded in the pairwise energy terms of the dispersion correction (see Table S3). The lattice energy represents the binding energy of the methanol molecules in the crystal and is defined as the sublimation energy of the methanol molecules without corrections for the zero-point vibrational energy (ZPVE) and the quantum nature of the OH proton.

D3 correction	a (Å)	b (Å)	c (Å)	V (Å^3)	E_b (eV)
all atoms	4.901	4.417	8.870	192.0	0.595
OH groups excluded	4.949	4.423	8.960	196.1	0.542
only Me groups included	5.068	4.426	9.127	204.7	0.474
no dispersion correction	5.274	4.454	9.269	217.7	0.398

Table S3. Optimized lattice parameters and lattice energy of solid methanol in the α-phase for different choices of atoms included/excluded in the Grimme D3 dispersion correction. V is the volume of the unit cell.

Comparison of the calculated lattice parameters with the experimental values already indicates that some degree of dispersion correction is needed to get a reasonable agreement for the structure of solid methanol. The experimental sublimation enthalpy for solid methanol in the α-phase is 0.486 eV (46.9 kJ/mol).[9] This value includes the ZPVE contributions, which have to be removed before we can compare it to the calculated lattice energies in Table S3. Vibrational frequencies of methanol molecules in the gas phase and in the solid

were calculated by a finite difference scheme. For the solid we restricted the calculation to the Γ-point phonons of the unit cell of the α-phase (66 normal modes for the unit cell with four methanol molecules). The ZPVE correction can be decomposed into two contributions: the first stems from the reduction of the frequencies of the internal methanol vibrations (12 normal modes) due to H-bonding, which reduces the ZPVE of the solid compared to the gas-phase molecule. However, this reduction is overcompensated by the contributions of the frustrated rotations and translations in the solid. For the gas-phase methanol molecule we obtain a ZPVE of 1.349 eV (1.351 eV when the experimental gas-phase vibrational frequencies are used), and 1.412 eV/molecule for the solid. Thus, the solid has a 0.063 eV higher ZPVE than the gas-phase molecule. This value has to be added to the experimental sublimation enthalpy of 0.486 eV to arrive at the experimental lattice energy of 0.549 eV.

If we compare this value to the DFT-calculated lattice energies in Table S3 we see that the D3 correction with all atoms included overbinds. The best agreement is found if the OH group is excluded from the D3 dispersion correction, as already expected from the D3 benchmark calculations listed in Ref. [8]. In fact, with this choice we find an excellent agreement for the lattice energy between theory and experiment, with a value of 0.542 eV compared to 0.549 eV, respectively. Also the calculated volume of the unit cell shows reasonable agreement with experiment. On the other hand, without any dispersion correction, the structure and the lattice energy of solid methanol cannot be described in a reliable way. Therefore, in our calculations for methanol molecules on the $In_2O_3(111)$ surface we included only the molecule–molecule interaction between the methoxy units in the Grimme D3 dispersion scheme, but excluded the OH groups.

Please note, in Table 1 of the manuscript we report the experimental multilayer desorption energy E_d of 0.475 eV for the α desorption peak at ≈120 K in the TPD spectrum of Figure 1 together with the calculated lattice energy E_b of 0.542 eV for solid methanol. The measured desorption energy E_d includes the ZPVE with respect to the gas phase, but the lattice energy E_b does not. Therefore, E_d has to be compared to the sublimation enthalpy (0.486 eV at 145 K)[9] and E_b to the ZPVE-corrected sublimation energy of 0.549 eV (see above).

For the different methanol structures on $In_2O_3(111)$, the value of 0.063 eV can be taken as an upper limit for the ZPVE correction of the binding energies reported in Table 1. On a surface, the frequencies of the frustrated rotations and translations of the methanol molecules are reduced compared to the solid, which lowers the ZPVE correction. The ZPVE correction can even change sign and result in a small increase in the reported binding energy by the ZPVE.

11) Atomic structure of the adsorbate-free surface

The primitive (1×1) unit cell of the bulk-terminated $In_2O_3(111)$ surface contains an O–In–O trilayer consisting of 16 In and 24 O atoms (see Figure 1 in the main text). The unit cell has 3-fold symmetry. The positions of the symmetry axes are labeled 'A', 'B', and 'C'. Four of the 16 In atoms are 6-fold coordinated In(6c) in two symmetry-inequivalent positions (labeled 'b' and 'd'), and 12 atoms are 5-fold coordinated In(5c) in four symmetry-inequivalent positions (labeled 'a', 'c', 'e' and 'f'). Half of the 24 O atoms sit above the In layer and are 3-fold coordinated O(3c), and the other half are 4-fold coordinated O(4c) with a position below the In layer. The 12 O(3c) atoms occupy four symmetry-inequivalent sites, labeled 'α', 'β', 'γ', and 'δ' in Figure 1 of the main text. In Figure S9 we have introduced additional labels to a few more surface atoms to allow for an unambiguous notation of all probed configurations of methanol molecules in the (1×1) unit cell of the $In_2O_3(111)$ surface, see the following sections.

In Table 1 of the manuscript we report *differential* binding energies per molecule:

$$E_\mathrm{b} = \Big(E_\mathrm{slab}(N_i) + \big(N_f - N_i\big)\, E_\mathrm{mol}^\mathrm{MeOH} - E_\mathrm{slab}(N_f)\Big)/\big(N_f - N_i\big) \quad .$$

$E_\mathrm{slab}(N_i)$ and $E_\mathrm{slab}(N_f)$ are the total energies of the slabs with the initial and final number of methanol molecules N_i and N_f, respectively, and $E_\mathrm{mol}^\mathrm{MeOH}$ is the total energy of the methanol gas-phase molecule. In the following sections, however, we will also report *average* binding energies per molecule:

$$\overline{E}_\mathrm{b} = \Big(E_\mathrm{slab}(0) + N_f\, E_\mathrm{mol}^\mathrm{MeOH} - E_\mathrm{slab}(N_f)\Big)/N_f \quad .$$

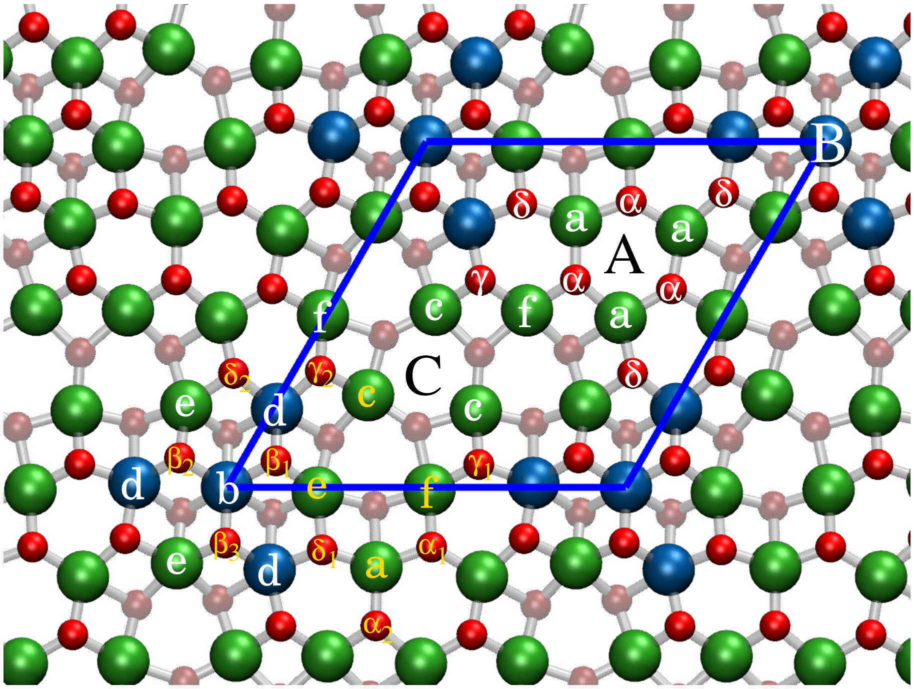

Figure S9. Structure of the bulk-terminated $In_2O_3(111)$ surface. In(6c) is shown in blue, In(5c) in green, O(3c) in bright red and O(4c) in shaded red. The surface unit cell is indicated by solid blue lines. The 3 high-symmetry positions of the surface with a 3-fold rotational symmetry axis, *i.e.*, the center of the In(5c)–O(3c) rings, the central In(6c) atom and the center of the In(5c)–O(4c) rings, are labeled by A, B and C, respectively. Labels a–f and α–δ indicate symmetry-equivalent positions of the 16 surface In and the 12 O(3c) atoms (see text). Additional subscripts have been added to discriminate symmetry-equivalent O(3c) atoms for an exact description of all methanol adsorption sites considered in the DFT calculations. The atomic positions listed in the forthcoming tables are highlighted in yellow.

12) Adsorption of single methanol molecules

Molecular adsorption: On oxides, methanol molecules coordinate in their most stable configuration with their O_M oxygen atom to an undercoordinated surface cation via one of their electron lone pairs. In our systematic search for the most favorable adsorption site for undissociated methanol molecules on the $In_2O_3(111)$ surface we placed a single methanol molecule in various orientations with its O_M atom on-top of all inequivalent In(5c) sites in the (1×1) unit cell and started a geometry optimization, see Table S4. After relaxation, the methanol molecule sits slightly tilted on the surface with the H_M of its OH group oriented toward a 3-fold coordinated surface O(3c) atom. In Table S4, the final orientation of the adsorbed methanol molecule is given by the label of the surface O(3c) to which the H_M is pointing and the position of the methyl (Me) group will be described either to be in clockwise ('CW') or counterclockwise ('CCW') orientation with respect to the H_M.

position of O_M (surface In atom)	orientation of H_M (surface O atom)	orientation of Me	E_b (eV)
ontop-a	α_2	CW	0.49
ontop-a	α_1	CW	0.54
ontop-a	δ_1	CCW	0.59
ontop-c	γ_2	CW	0.64
ontop-c	γ_2	CCW	0.68
ontop-e	β_1	CW	0.61
ontop-e	δ_1	CW	0.64
ontop-e	β_1	CCW	0.75
ontop-f	γ_1	CW	0.66
ontop-f	α_1	CCW	0.69

Table S4. Structure and binding energy E_b for molecular adsorption of a single methanol molecule in the primitive (1×1) unit cell of the $In_2O_3(111)$ surface. The position of the methyl group (Me) is either in clockwise ('CW') or counterclockwise ('CCW') orientation with respect to its H_M.

Dissociative adsorption: For the methoxy group we considered all symmetry-inequivalent on-top positions above single In(5c) atoms and all possible bridging sites between two neighboring In(5c), see Table S5. The proton H_M from methanol dissociation was placed on-top of all possible 3-fold coordinated O(3c) atoms. In all cases, the highest binding energy was found if the H_M occupies an O(3c) site that shares an In(5c) with the methoxy group. Therefore, all configurations, in which the H_M is farther away, have been omitted in Table S5 (see also Table S6).

position of O_M (surface In atom)	position of H_M (surface O atom)	E_b (eV)
ontop-a	δ_1	0.49
ontop-a	α_1	0.42
ontop-a	β_1	0.29
ontop-c	β_1	unstable$^{(1)}$
ontop-c	δ_2	0.55
ontop-d	β_1	0.15
ontop-e	δ_1	0.62
ontop-e	β_1	unstable$^{(2)}$
ontop-f	α_1	0.70
ontop-f	γ_1	0.44
bridge-ce	β_1	1.17
bridge-ce	γ_2	0.37
bridge-cf		unstable$^{(2)}$
**bridge-ef**	β_1	**1.22**
bridge-ef	β_2	1.14
bridge-ef	δ_1	1.01
bridge-ef	α_1	0.64
bridge-ef	γ_1	0.57
bridge-ef	γ_2	0.31

$^{(1)}$ methoxy group relaxes into bridge-ce position

$^{(2)}$ methoxy group relaxes into bridge-ef position

Table S5. Structure and binding energy E_b for dissociative adsorption of a single methanol molecule in the primitive (1×1) unit cell of the $In_2O_3(111)$ surface.

Tables S4+S5 show that for single methanol molecules dissociation is much more favorable than molecular adsorption. The In(5c) site 'a' is clearly less stable than positions 'c', 'e' and 'f', for molecular as well as dissociative adsorption. Therefore, site 'a' will be no longer considered in the forthcoming calculations. The highest binding energy is found for the configuration in which the proton adorbs on a 'β' site (the surface oxygen atom with the highest proton affinity[2]) and the methoxy group occupies the neighboring 'bridge-ef' position. Since there are three 'bridge-ef' and three 'β' sites in the primitive (1×1) unit cell, up to three methanol molecules can be adsorbed in this configuration.

The average binding energies when one, two or three methanol molecules occupy the 'bridge-ef' and the 'β' sites in a (1×1) unit cell are given in Table S6. The results show that the dissociated proton clearly preferes to be on a neighboring O(3c) site to the methoxy group. The binding energy decreases even if the H_M still occupies a 'β' site but not the one that shares an In(5c) with the methoxy group. Interestingly, the binding energy per molecule decreases with increasing methanol coverage. The reason is that the dissociative adsorption of the methanol molecules induces a significant re-relaxation of the surface. Upon cleavage, the surface lowers its energy by 3.22 eV per unit cell by relaxation. These relaxations are partly reverted upon the adsorption of molecules, which contributes significantly to the overall binding energy. However, the re-relaxation energy is available to the full extent only for the first molecule and is reduced for the second and third methanol (see main text).

number of methanol molecules	position of O_M (surface In atom)	position of H_M (surface O atom)	$\overline{E}_b$ (eV)
1	bridge-ef	β_1	1.22
		β_2	1.14
		β_3	1.10
2	bridge-ef	β_1, β_2	1.17
		β_1, β_3	1.11
3	bridge-ef	β_1, β_2, β_3	1.11

Table S6. Binding energies $\overline{E}_b$ per molecule for occupying the three symmetry-equivalent adsorption sites 'bridge-ef' and 'β' in the primitive (1×1) unit cell of the $In_2O_3(111)$ surface by one, two or three methanol molecules.

13) Adsorption of methanol pairs

The structures listed in Table S6 are likely to be the most favorable configurations for two and three methanol molecules in the primitive (1×1) surface unit cell. However, when methanol molecules form pairs, the formation of H-bonds can significantly stabilize alternative structures. Therefore, to make sure that we are not missing such a new motif in our systematic search, we probed all possible configurations in which the second methanol molecule does not occupy the second 'bridge-ef/β' site. Only methanol pairs on nearest-neighbor positions were considered to capture the stabilizing effect of H-bond formation. We start our structure search with the first methanol molecule in the 'bridge-ef/β' position. However, we allow that the second molecule displaces the methoxy group from its bridge site. For this second methanol molecule, disocciative (see Table S7) as well as molecular (see Table S8) adsorption are considered.

first O_M	first H_M	second O_M	second H_M	$\overline{E}_b$ (eV)
bridge-ef	β_1	ontop-c	γ_2	0.66
bridge-ef	β_1	ontop-a	α_1	0.77
bridge-ef	β_1	ontop-a	δ_1	0.78
bridge-ef	β_1	ontop-a	γ_1	0.37
bridge-ef	β_1	hollow-C	δ_1	0.89
bridge-ef	β_1	hollow-C	γ_1	0.82
bridge-ef	β_1	hollow-C	δ_2	0.79
bridge-ef	β_1	hollow-C	γ_2	0.64

Table S7. Structure and binding energy $\overline{E}_b$ per molecule for two dissociated methanol molecules in the primitive (1×1) unit cell of the $In_2O_3(111)$ surface.

The results of Tables S7+S8 show that the configuration of Table S6 remains the most favorable structure for two methanol molecules in the primitive unit cell. However, when not occupying simultaneously two 'bridge-ef' and two 'β' sites, two dissociated molecules now become less favorable than mixed dissociated/molecular structures. The most favorable alternative structural motif (a dissociated molecule at site 'ontop-e/β' and an intact molecule at site 'ontop-c' with CW orientation) can be placed three times in the primitive (1×1) surface unit cell. This gives the structure with the highest binding energy for 6 methanol molecules in the primitive unit cell that we found in our DFT calculations, see Figure S10.

diss: O_M	mol: O_M	mol: orient	$\overline{E}_b$ (eV)
ontop-e	ontop-c	CW	1.05
ontop-e	ontop-c	CCW	1.03
ontop-e	ontop-f	CCW	1.01
ontop-e	ontop-a	CW	0.84
ontop-e	ontop-a	CW	0.83
bridge-ef	ontop-a	CCW	0.83
ontop-f	ontop-a	CCW	0.84

Table S8. Structure and binding energy $\overline{E}_b$ per molecule for two methanol molecules with mixed adsorption mode in the primitive (1×1) unit cell of the In_2O_3(111) surface. The first molecule is dissociated. The proton H_M always occupies site 'β_1'. The second methanol molecule remains intact and forms an H-bond with its H_M to the O_M of the methoxy group of the dissociated molecule. The position of the methyl group of the second molecule is either in clockwise ('CW') or counterclockwise ('CCW') orientation with respect to its H_M.

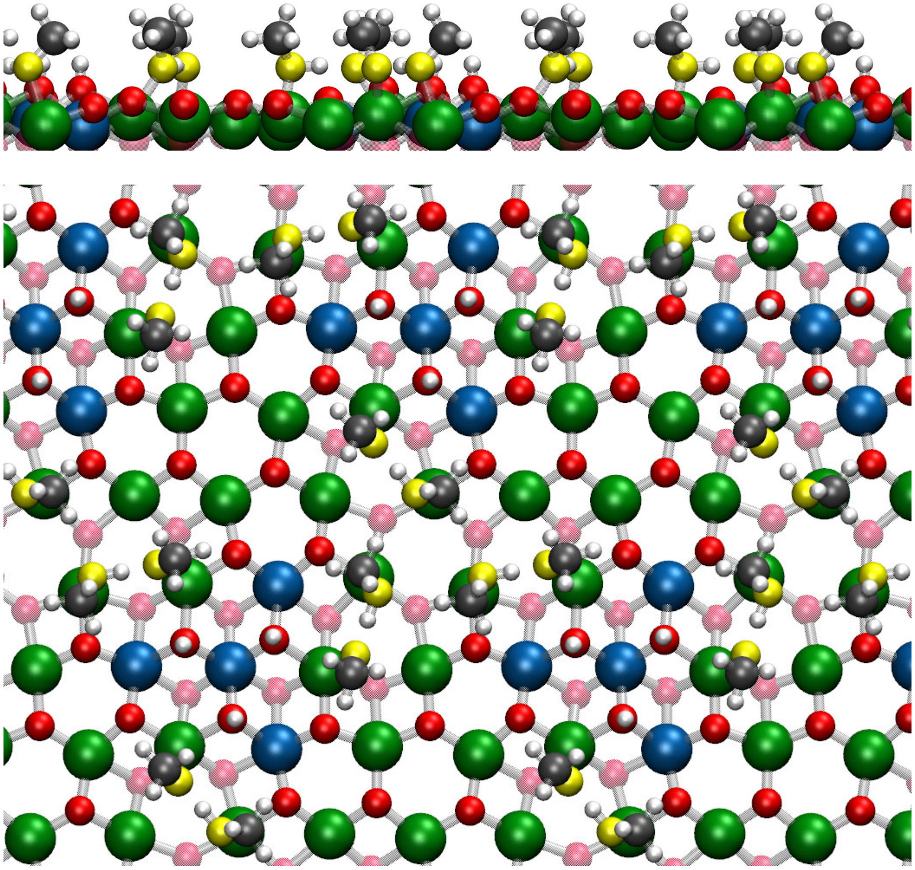

Figure S10. Side and top view of the most favorable structure with 6 methanol molecules in the (1×1) unit cell of the In_2O_3(111) surface. Three molecules are dissociated and occupy the 'ontop-e' and 'β' sites, the other three molecules remain intact and sit on-top of 'c' atoms (the first configuration of Table S8 is tripled and relaxed).

14) Adsorption of methanol trimers

Next, we explore all possibilities in which three methanol molecules occupy three neighboring inequivalent In(5c) sites 'c', 'e' and 'f'. Since on these sites mixed molecular/dissociated structures are already more stable for two molecules than full dissociation, we considered only configurations with one dissociated and two intact methanol molecules. Since now only three adsorption sites are available for the three molecules, the methoxy group of the dissociated methanol molecule has to be placed in an on-top position, see Table S9.

Surprisingly, in the most favorable configuration the methoxy group has moved to the 'ontop-f' site but not to 'ontop-e', which would be the neighboring position to its dissociated proton at 'β'. Tripling the most favorable alternative configuration (a dissociated molecule at site 'ontop-f/β' and two intact molecules at sites 'ontop-c' and 'ontop-e' with CW orientation) gives the best structure for 9 methanol molecules in the primitive unit cell that we found in our DFT calculations, see Figure S11.

diss: O_M	mol1: O_M	mol1: orient	mol2: O_M	mol2: orient	$\overline{E}_b$ (eV)
ontop-f	ontop-c	f, CW	ontop-e	f, CW	1.00
ontop-f	ontop-c	f, CW	ontop-a	f, CCW	0.90
ontop-e	ontop-c	e, CW	ontop-f	e, CW	0.92
ontop-e	ontop-c	e, CW	ontop-a	e, CCW	0.85
ontop-c	ontop-e	c, CW	ontop-f	c, CW	0.92
ontop-c	ontop-e	f, CW	ontop-f	c, CW	unstable$^{(1)}$
ontop-c	ontop-e	c, CW	ontop-c2	c, CCW	0.88
ontop-c	ontop-f	c, CW	ontop-c2	c, CCW	0.99
ontop-c	ontop-e	c, CW	ontop-c3	c, CCW	0.99
ontop-c	ontop-f	c, CW	ontop-c3	c, CCW	0.99

$^{(1)}$ mol2 dissociates and proton is transfered to the methoxy group

Table S9. Structure and binding energy $\overline{E}_b$ per molecule for three methanol molecules with mixed adsorption mode in the primitive (1×1) unit cell of the $In_2O_3(111)$ surface. The first molecule is dissociated. Its proton H_M always occupies site 'β_1'. The second and third methanol molecule remain intact and always form an H-bond to the O_M of a neighboring dissociated or undissociated methanol molecule. The position of the methyl group is either in clockwise ('CW') or counterclockwise ('CCW') orientation with respect to its H_M.

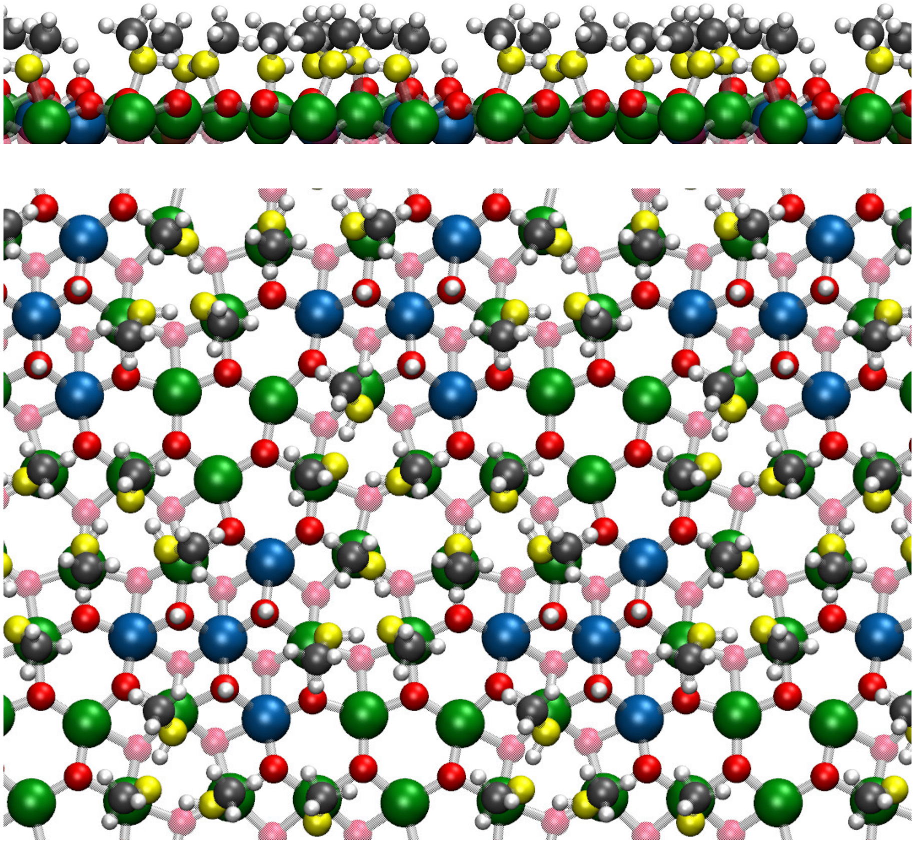

Figure S11. Side and top view of the most favorable structure with 9 methanol molecules in the (1×1) unit cell of the $In_2O_3(111)$ surface. Three molecules are dissociated and occupy the 'ontop-f' and 'β' sites, the other six molecules remain intact and sit on-top of 'c' and 'e' atoms (the first configuration of Table S9 is tripled and relaxed).

15) Structures with 12 adsorbed methanol molecules (β phase)

Finally, we extended our search for the lowest-energy structures to 12 methanol molecules on the $In_2O_3(111)$ surface. First we considered that 3 more molecules adsorb on the still available 'a' sites of the best structure with 9 molecules per unit cell shown in Figure S11 (η phase). If we enforce the 3-fold symmetry of the surface, we find only two stable structures in which the H_M atom either points toward a neighboring 'δ' or 'α' site (see Figure S12a,b). Structures in which the H_M points away from the surface or forms a H bond to another methanol molecule were unstable and the three additional methanol molecules around site A desorbed from the surface in the structure optimization. The methyl groups can adopt only one orientation due to steric restrictions in the now rather crowded molecular layer.

Next, we broke the 3-fold symmetry and considered mixed orientations of the three methanol molecules around site A, with the H_M pointing towards site 'α', 'δ', or an O_M of a neighboring molecule. The best structure with a somewhat larger binding energy of the three molecules than in the symmetric case is shown in Figure S12c. In the last step we allowed one of the additional molecules to dissociate. After testing different molecular orientations and different positions of the methoxy group and the proton of the dissociated molecule we found Figure S12d as the lowest-energy structure. However, even in this best structure, the binding energy of the last three methanol molecules is only 0.40 eV, significantly less than the calculated lattice energy of solid methanol of 0.542 eV (see Section 10). This indicates that the growth of methanol clusters and solid methanol is favored over the adsorption in area A at the sites 'a'.

In our next attempt we placed the three additional methanol molecules on-top of the O_SH groups formed by the dissociation of the three methanol molecules in the η configuration of Figure S11 with 9 methanol molecules per surface unit cell. We enforced the 3-fold symmetry and established H-bonds between the molecules (ring motif, see Figure S13a). In this structure the binding energy for the three on-top methanol molecules is already higher than in the best structure with adsorption at the 'a' sites in Figure S12. However, the binding energy of 0.49 eV per molecule is still smaller than the lattice energy of solid methanol.

For an unbiased search of structures with even lower energy, for example, by breaking the symmetry, rearranging the H-bond network, or lifting the structure motif of the η configuration (see golden circles in Figure S13) we turned to *ab initio* molecular dynamics simulations and a simulated annealing approach. By this procedure, as described in the manuscript, we could identify one unique new structure with a methanol binding energy of 0.58 eV for the last three molecules (Figure S13b). The second-best structure we found by this procedure is the ring motif shown in Figure S13a.

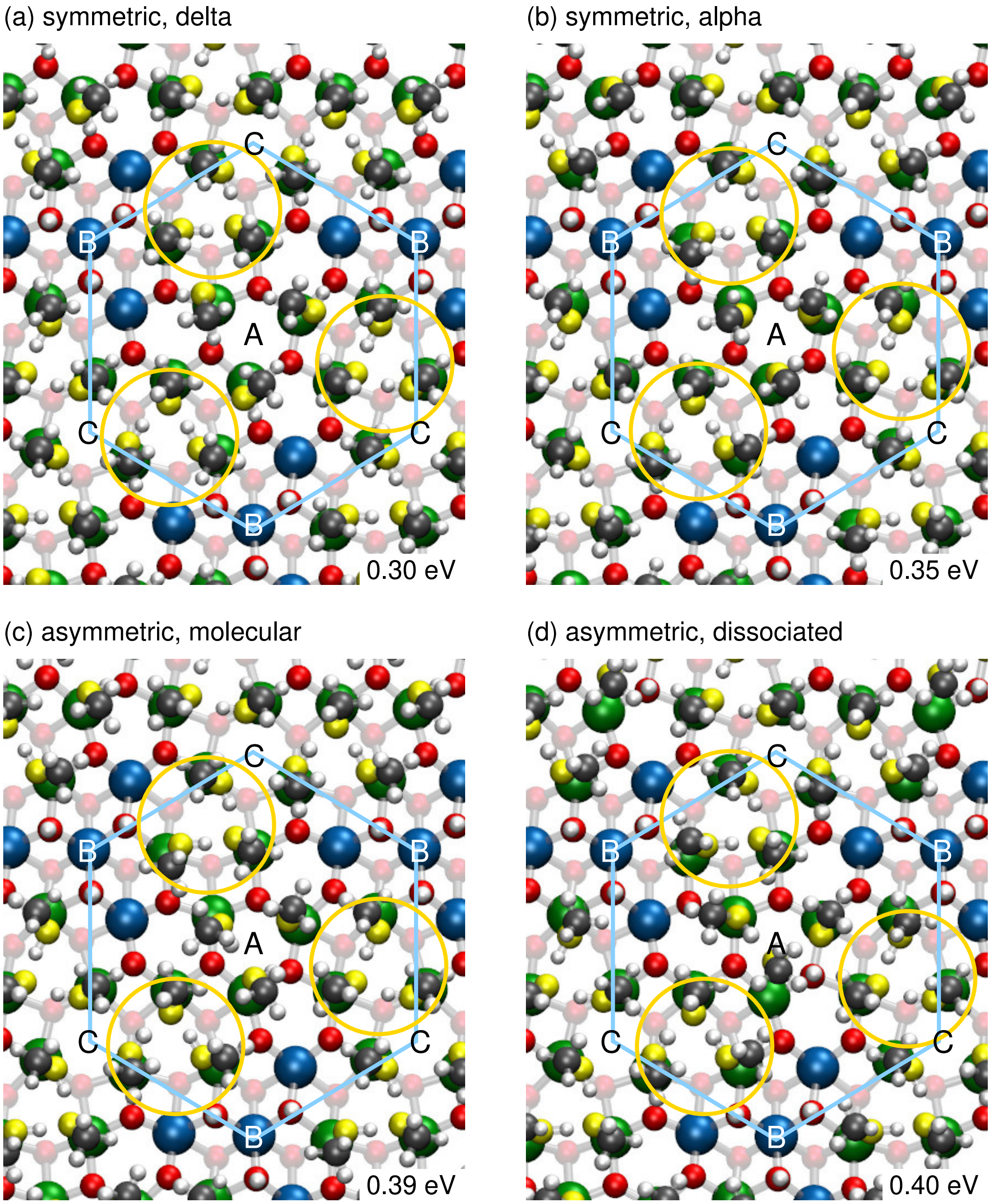

Figure S12. Representative low-energy structures of 12 methanol molecules in the (1×1) unit cell of the $In_2O_3(111)$ surface with 3 methanol around site A (adsorbed on 'a' sites) and 9 methanol adopting the structure of the η phase shown in Figure S11. A Wigner-Seitz unit cell, centered around site A, is shown by a solid light-blue line. The structure motif of the η phase (a dissociated molecule at site 'ontop-f/β' and two intact molecules at sites 'ontop-c' and 'ontop-e') is marked by golden circles. The *differential* binding energy of the three additional molecules is given in the bottom right corner of each panel.

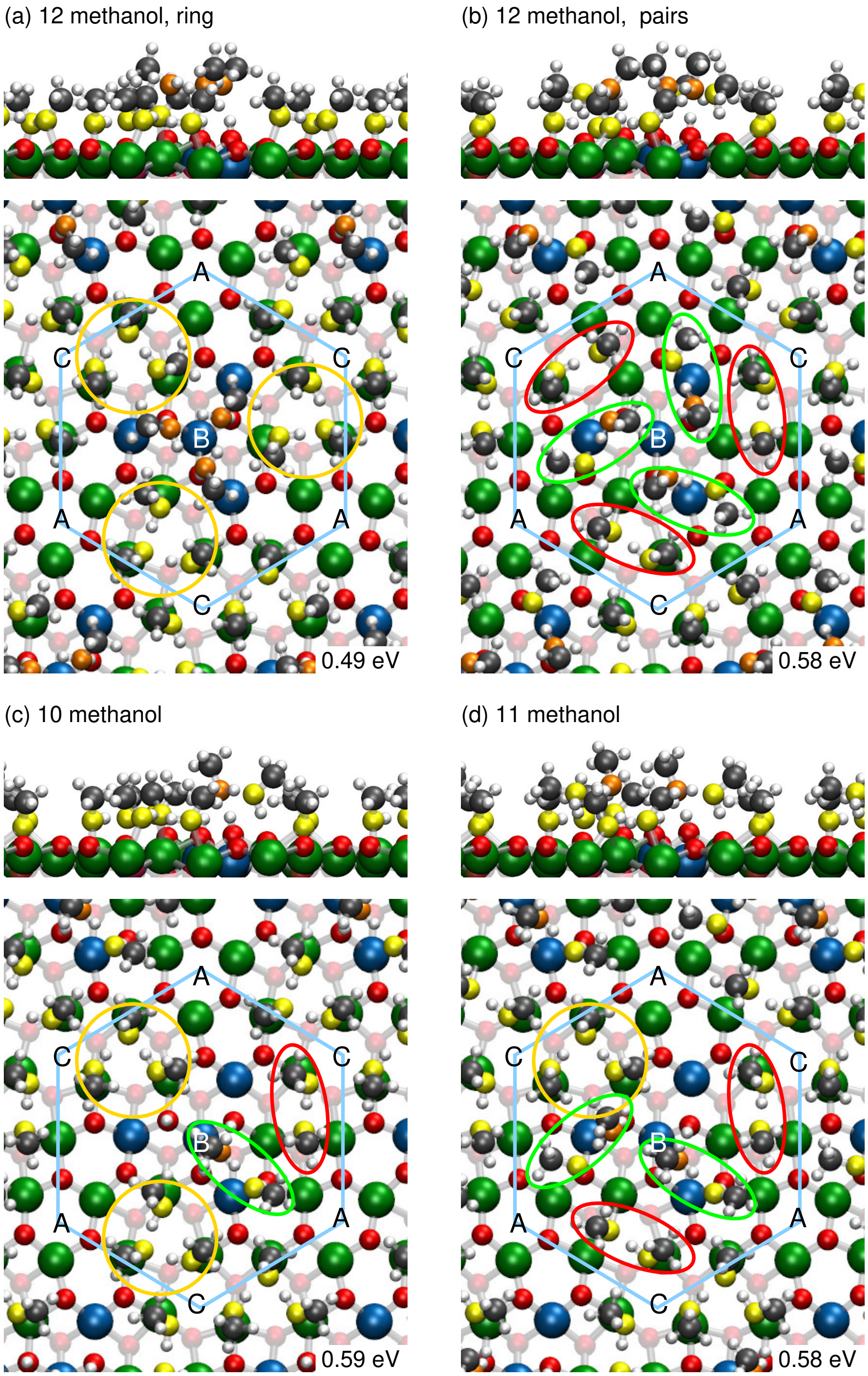

Figure S13. Representative structures with methanol molecules above O_SH groups (indicated by orange O_M atoms). (a,b) Lowest-energy structures with 12 molecules, (c,d) structures after removing two and one on-top molecules from (b), respectively. The solid light-blue line shows the B-centered Wigner-Seitz unit cell. Golden circle: structure motif of the η phase; green ellipse: pair of a MeOH on-top of O_SH and a MeOH lifted from its former 'e' site; red ellipse: pair of a dissociated MeOH at 'bridge-ef/β' and an intact MeOH at site 'c'. The *differential* binding energy of the on-top methanol molecules is given in the bottom right corner of each panel.

16) Surface phase diagram

The binding energies per molecule of Table 1 in the main manuscript can be converted into energy gains per surface area upon methanol adsorption.[10] The result is the surface phase diagram shown in Figure S14. Each of the discussed structures appears as a thermodynamically stable phase for an interval of the methanol chemical potential (*i.e.*, a defined temperature and pressure range). Please note, ZPVE contributions and vibrational and configurational entropy contributions at finite temperature are not included in this phase diagram. These corrections are not expected to lead to any qualitative changes of the phase diagram, they will only modify the exact $\Delta\mu_M$ values of the phase boundaries.

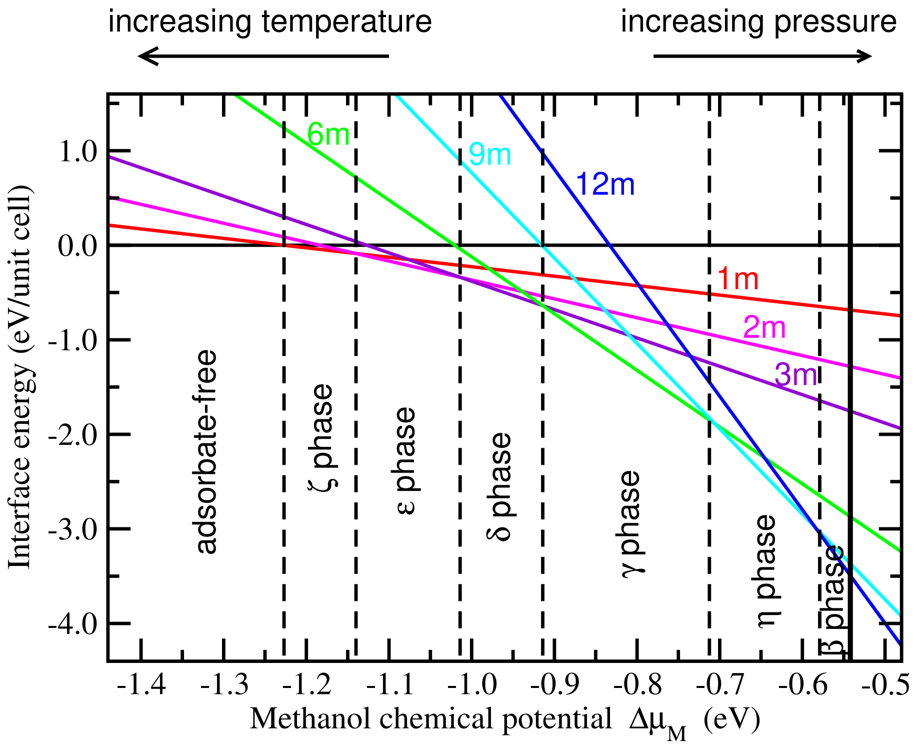

Figure S14. Surface phase diagram of the $In_2O_3(111)$ surface as a function of the methanol chemical potential $\Delta\mu_M$. The label 'xm' gives the surface coverage in number of methanol molecules per surface unit cell. The vertical dashed lines separate different phases and the solid black line at -0.542 eV indicates the lattice energy of solid methanol.

References

[1] M. Wagner, P. Lackner, S. Seiler, A. Brunsch, R. Bliem, S. Gerhold, Z. Wang, J. Osiecki, K. Schulte, L.A. Boatner, M. Schmid, B. Meyer, U. Diebold, Resolving the Structure of a Well-ordered Hydroxyl Overlayer on In_2O_3(111): Nanomanipulation and Theory, *ACS Nano* **2017**, *11*, 11531–11541; DOI: 10.1021/acsnano.7b06387

[2] M. Wagner, B. Meyer, M. Setvin, M. Schmid, U. Diebold, Direct assessment of the acidity of individual surface hydroxyls, *Nature* **2021**, *592*, 722–725; DOI: 10.1038/s41586-021-03432-3

[3] H. Chen, M.A. Blatnik, C.L. Ritterhoff, I. Sokolović, F. Mirabella, G. Franceschi, M. Riva, M. Schmid, J. Čechal, B. Meyer, U. Diebold, M. Wagner, Water Structures Reveal Local Hydrophobicity on the In_2O_3(111) Surface, *ACS Nano* **2022**, *16*, 21163–21173; DOI: 10.1021/acsnano.2c09115

[4] A. Wolfram, M. Muth, J. Köbl, A. Mölkner, S. Mehl, N. Tsud, H.-P. Steinrück, B. Meyer, O. Lytken, Phenylphosphonic Acid on Rutile TiO_2(110): Using Theoretically Predicted O-1s Spectra to Identify the Adsorption Binding Modes, *J. Phys. Chem. C* **2024**, *128*, 12735–12753; DOI: 10.1021/acs.jpcc.4c03690

[5] S. Tanuma, C.J. Powell, D.R. Penn, Calculations of electron inelastic mean free paths. V. Data for 14 organic compounds over the 50–2000 eV range, *Surface and Interface Analysis* **1994**, *21*, 165–176; DOI: 10.1002/sia.740210302

[6] B.H. Torrie, S.-X. Weng, B.M. Powell, Structure of the α-phase of solid methanol, *Mol. Phys.* **1989**, *67*, 575–581; DOI: 10.1080/00268978900101291

[7] B.H. Torrie, O.S. Binbrek, M. Strauss, I.P. Swainson, Phase Transitions in Solid Methanol, *J. Solid State Chem.* **2002**, *166*, 415–420; DOI: 10.1006/jssc.2002.9615

[8] S. Grimme, J. Antony, S. Ehrlich, H. Krieg, A consistent and accurate ab initio parametrization of density functional dispersion correction (DFT-D) for the 94 elements H–Pu; *J. Chem. Phys.* **2010**, *132*, 154104; DOI: 10.1063/1.3382344

[9] S. Lucas, D. Ferry, B. Demirdjian, J. Suzanne, Vapor Pressure and Solid Phases of Methanol below Its Triple Point Temperature, *J. Phys. Chem. B* **2005**, *109*, 18103–18106; DOI: 10.1021/jp053313v

[10] B. Meyer, H. Rabaa, D. Marx, Water adsorption on ZnO($10\bar{1}0$): from single molecules to partially dissociated monolayers, *Phys. Chem. Chem. Phys.* **2006**, *8*, 1513–1520; DOI: 10.1039/b515604a
